# AgriPath: a robust multi-objective path planning framework for agricultural robots in dynamic field environments

**DOI:** 10.3389/fpls.2025.1687747

**Published:** 2025-11-27

**Authors:** Chenghan Yang, Dingkun Zheng, Siming Chen, Madina Mansurova, Baurzhan Belgibaev, Baidong Zhao

**Affiliations:** 1 Faculty of Information Technology, Al-Farabi Kazakh National University, Almaty, Kazakhstan; 2 School of Data Science, Fudan University, Shanghai, China

**Keywords:** robotics, whale optimization algorithm, path planning, multi-objective optimization, precision agriculture

## Abstract

Robot path planning is a cornerstone of precision agriculture, enabling safe and efficient operations for agricultural robots. However, complex field environments—characterized by static and dynamic obstacles, dense vegetation, and unstructured terrain—pose significant challenges to effective path planning. Conventional methods, such as A*, Dijkstra, and rapidly exploring random tree (RRT), exhibit limitations in efficiency and adaptability to dynamic conditions. To address these challenges, this study introduces AgriPath, a robust multi-objective path planning framework that integrates an improved convolutional neural network (CNN), an improved A* algorithm, and an improved whale optimization algorithm (IWOA) to optimize pathfinding, convergence efficiency, and obstacle avoidance in complex agricultural settings. Key innovations include an improved CNN leveraging causal convolution and multi-head self-attention mechanisms to improve temporal modeling for short-term trajectory prediction, augmented by Gaussian perturbations to enhance initial solution diversity; an improved A* algorithm incorporating dynamic heuristic functions based on Normalized Difference Vegetation Index (NDVI), combined with Kalman filtering, to bolster global path adaptability; IWOA employing non-linear convergence factors and differential evolution mechanisms to dynamically balance path length, smoothness, and planning time; and an improved Douglas–Peucker algorithm paired with cubic B-spline smoothing and navigation command modules to ensure path simplification and real-time execution. Experiments conducted in the Modern Agricultural Demonstration Zone at Chengdu, Sichuan Province, China, across simple, moderate, and complex scenarios, demonstrate that AgriPath outperforms advanced algorithms—SBREA*, Ant Colony A*, Orchard A*, and Greedy A*—in path length, smoothness, planning time, and dynamic obstacle avoidance success rate, indicative of superior multi-objective optimization balance. This study significantly enhances the efficiency and robustness of agricultural robot path planning, offering a more adaptive solution for autonomous navigation in precision agriculture while providing new theoretical and practical directions for the field of path planning.

## 1 Introduction

As global agriculture continues its evolution toward greater intelligence and precision, agricultural robots have come to occupy a central role in field tasks such as crop inspection, autonomous spraying, precision fertilization, and pest and disease identification (Yang et al., 2024a; Yang et al., 2025; Zhao et al., 2025). High-density crop cultivation environments—characterized by narrow operating spaces and severe occlusions—demand heightened efficiency, robustness, and intelligent decision-making capabilities from robotic path planning systems (Padhiary et al., 2024). However, real-world farmlands are inherently unstructured. They are not only populated by dynamic obstacles such as moving workers, operating machinery, and rain-formed puddles, but also complicated by uneven terrain, dense vegetation, and sensing uncertainties (Shamshiri et al., 2024). Consequently, developing path optimization methods equipped with environmental perception, multimodal data fusion, and dynamic responsiveness has become a critical frontier for enhancing the autonomy of agricultural robots (Guo et al., 2024).

Extensive research efforts have been devoted to robotic path planning in agricultural contexts, yet existing algorithms remain constrained in their performance within complex farmland environments (Zhang and Li, 2023; Yang et al., 2024b). Graph search-based methods such as the Dijkstra algorithm guarantee a globally optimal path by exhaustively traversing all nodes—an approach theoretically well-suited to static terrains (Fedorov et al., 2025). However, in dense agricultural settings, its computational complexity grows exponentially, rendering it unsuitable for real-time applications, especially when dealing with large-scale grid maps or frequent path updates (Li et al., 2025; Liu et al., 2025c). Sampling-based methods, like the rapidly exploring random tree (RRT), exhibit a degree of adaptability to unstructured terrains via stochastic sampling and expansion strategies (Xu, 2024). Nonetheless, such methods often generate unsmoothed paths and, due to their reliance on randomness, can become trapped in local optima, resulting in overly long trajectories or conflicts with crop zones that ultimately impair operational efficiency (Reda et al., 2024; Liu et al., 2025a).

In contrast, the A* algorithm—merging heuristic search with grid modeling—has demonstrated strong applicability in agricultural contexts. It can efficiently produce near-optimal paths within structured grids, particularly excelling when static obstacle distributions are well-defined (Liu et al., 2023). However, its conventional implementation struggles in dynamically evolving environments, such as fields with moving workers, temporary puddles, or plant occlusions. The algorithm’s delayed path updates in such settings may result in elevated collision risks or diminished efficiency (Wang et al., 2024; Zhang et al., 2024c). To address these limitations, recent studies have pursued improvements to the A* algorithm by introducing dynamic obstacle prediction, heuristic function optimization, and multi-objective trade-offs (Zhang et al., 2022; Zheng et al., 2023). While these enhancements bolster performance in complex terrains, challenges remain in balancing real-time responsiveness with system robustness (Shen et al., 2024; Shen et al., 2025; Zhang et al., 2025). For instance, SBREA* improves global path generation by dynamically adjusting heuristic weights to better avoid static obstacles while also refining local planning for agile responses to moving obstacles (Xu et al., 2024). Ant Colony A* integrates principles of ant colony optimization, using pheromone-based strategies to enhance global path selection and enable local adaptation in high-density vegetation (Dai et al., 2019). Orchard A* focuses on orchard scenarios by refining grid resolution and heuristic design, improving global path quality, and optimizing local routes in unstructured and dynamic maize fields (Zhang et al., 2024a). Greedy A* prioritizes globally determined routes while selecting locally optimal segments to accelerate path updates and strengthen dynamic obstacle avoidance (Xiang et al., 2022). Nevertheless, these improved methods often fall short in achieving robust and real-time global–local collaborative planning. They tend to focus on single-objective optimization—typically path length—without adequately balancing multiple objectives such as path length, smoothness, and planning time; nor do they fully exploit multimodal perception and real-time execution capabilities. Thus, there is a pressing need for an integrated framework that unifies multimodal sensing, dynamic adaptive global–local planning, and multi-objective optimization to meet the challenges of complex field environments (Liu et al., 2025b; Yao et al., 2025).

To address the issues, this study introduces AgriPath—a multi-objective path planning framework tailored for agricultural robots operating in dynamic maize field environments. Experiments were conducted at the Modern Agriculture Demonstration Zone of Xihua University, Chengdu, Sichuan Province, China, encompassing three scenarios—simple, moderate, and complex—which simulate static, dynamic, and stochastic obstacle conditions, respectively. AgriPath integrates an improved convolutional neural network (CNN), an improved A* algorithm, and an improved whale optimization algorithm (IWOA) to realize precise short-term trajectory prediction, dynamically adaptive global path planning, and multi-objective optimization across path length, smoothness, and planning time.

The novelty of this research lies in the construction of a closed-loop system spanning from multimodal perception to real-time control. The core contributions are as follows:

1. An improved CNN module that boosts temporal sequence modeling through causal convolution and multi-head self-attention mechanisms,
2. An improved A* algorithm that employs dynamic heuristic functions and Kalman filtering to reinforce global path safety,
3. A multi-objective optimization framework utilizing non-linear convergence and differential evolution to efficiently balance competing goals, and
4. An improved Douglas–Peucker algorithm and a lightweight navigation instruction module that jointly ensure path simplification and real-time responsiveness.

These innovations collectively elevate the efficiency and robustness of agricultural robot navigation, offering a reliable solution for precision agriculture by significantly enhancing critical tasks such as spraying, inspection, and monitoring. The optimized paths ensure uniform pesticide application and comprehensive crop health assessment, while the robust framework supports real-time adaptability for fertilization and disease detection, opening new directions for both theoretical and applied research in path planning.

## 2 Materials and methods

### 2.1 Research location

**Figure 1:**
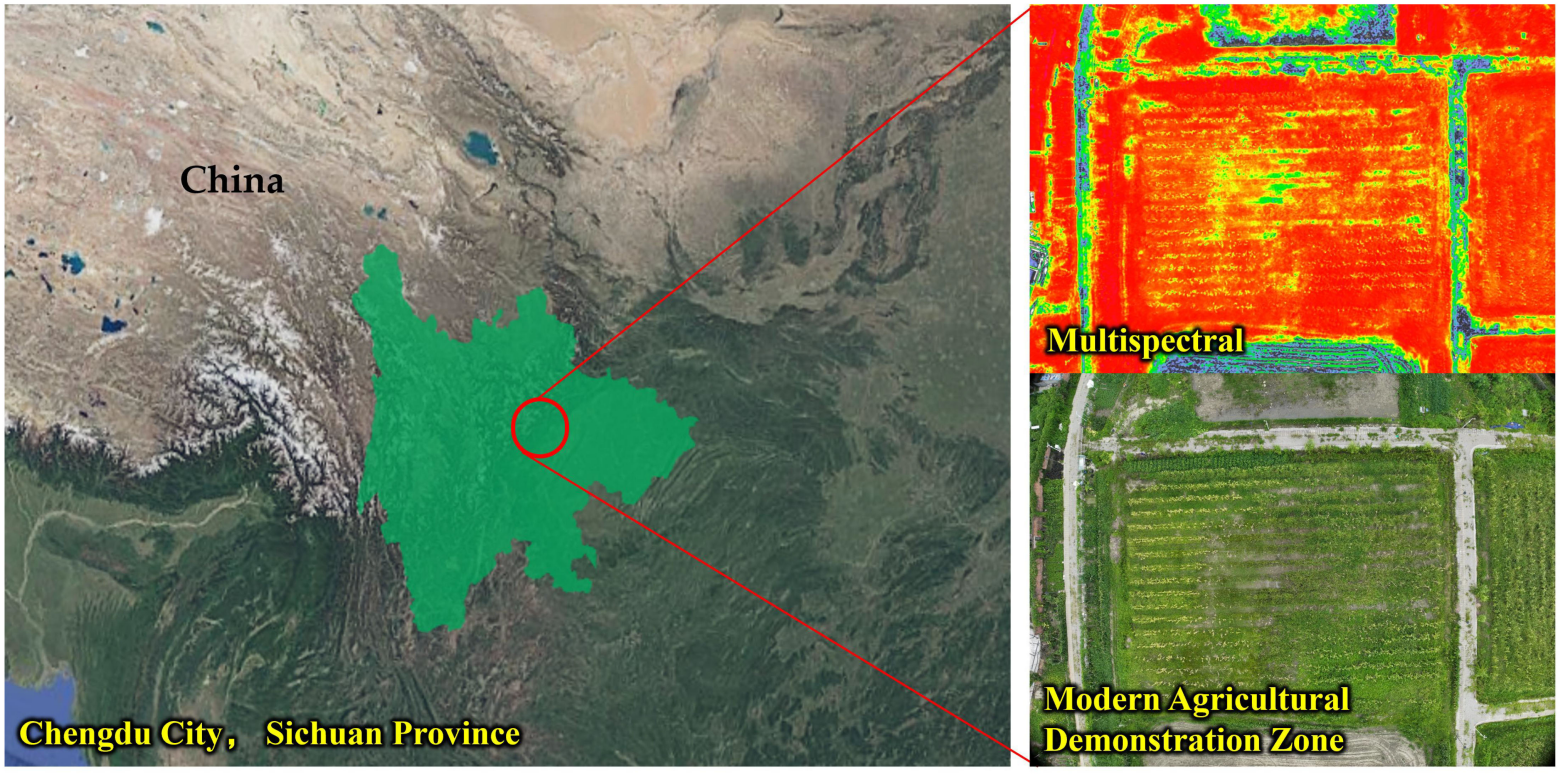
Modern agriculture demonstration zone, located in the suburban area of Chengdu, Sichuan Province, China.

This study was conducted at the Modern Agriculture Demonstration Zone of Xihua University, located in the suburban area of Chengdu, Sichuan Province, China (**Figure 1**). The site spans approximately 50 hectares and is primarily cultivated with maize, representing typical characteristics of plain farmlands. The terrain is generally flat with a slope of less than 3°. The demonstration area adopts a fully mechanized maize cultivation system, featuring a row spacing of 1.0 m and a plant spacing of 0.25 m. The maize plants reach heights between 1.8 and 2.5 m, with a growth cycle ranging from 90 to 120 days. This cycle encompasses critical operational phases from the vegetative stage to maturity.

### 2.2 A robust multi-objective path planning framework

**Figure 2:**
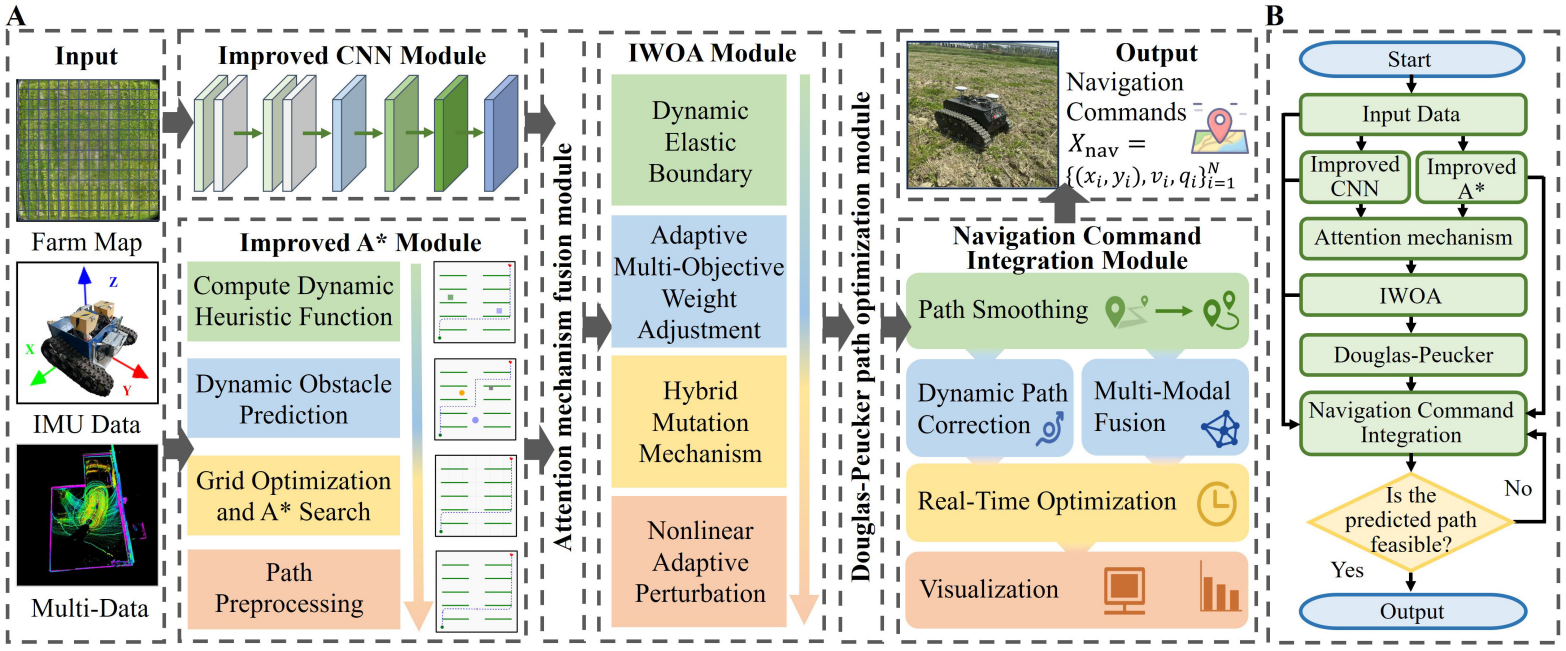
A robust multi-objective path planning framework. **(A)** Overall architecture of the framework. **(B)** Flowchart of the framework.

To address the complex and dynamic nature of maize field environments, this study proposes a comprehensive path planning and optimization framework for agricultural robots that integrates local trajectory prediction, global path planning, and multi-objective optimization (**Figure 2A**). The overall system architecture implements a closed-loop process from perception and decision-making to control. The improved CNN module predicts short-term local trajectories; the improved A* algorithm generates global paths; an attention mechanism fusion module synthesizes both local and global trajectories to form an initial path; the IWOA conducts multi-objective optimization; the improved Douglas–Peucker algorithm simplifies and smooths the path; and finally, the navigation instruction generation module converts the optimized path into executable commands for the robot, supporting dynamic obstacle avoidance and real-time adjustments (**Figure 2B**).

The overall model can be formally expressed as Equation 1:

(1) $$\left\{ \begin{array}{l} X_{CNN}^{t}=F_{CNN}(D_{fused},D_{IMU})\\ {\hat{X}}_{A*}=F_{A*}(M,D_{multi})\\ X_{init}=F_{Attention}(X_{CNN}^{t},{\hat{X}}_{A},D_{fused},M)\\ X_{best}=F_{IWOA}(X_{init},D_{fused},M)\\ X_{simplified}=F_{DP}(X_{best},D_{fused},M)\\ U_{nav}=F_{control}(X_{simplified},D_{fused}) \end{array}\right.$$

The definitions of the variables and functions in Equation 1 are provided in **Table 1**.

**Table 1:** Definitions of the variables and functions in the overall model.

Notation	Definition
$X_{\text{CNN}}^{t}$	Local short-term trajectory predicted by CNN module
${\hat{X}}_{A*}$	Global path generated by improved A* algorithm
$X_{\text{init}}$	Initial path after fusing path information
$X_{\text{best}}$	Multi-objective path optimized by IWOA
$X_{\text{simplified}}$	Path smoothed and simplified by Douglas–Peucker (DP) algorithm
$U_{\text{nav}}$	Final navigation control command
$D_{\text{fused}},D_{\text{IMU}},D_{\text{multi}}$	Multimodal fused data, inertial measurement unit (IMU) data, and multimodal real-time perception data, respectively
*M*	Cornfield environmental map data

CNN, convolutional neural network; IWOA, improved whale optimization algorithm.

#### 2.2.1 Improved convolutional neural network module

**Figure 3:**
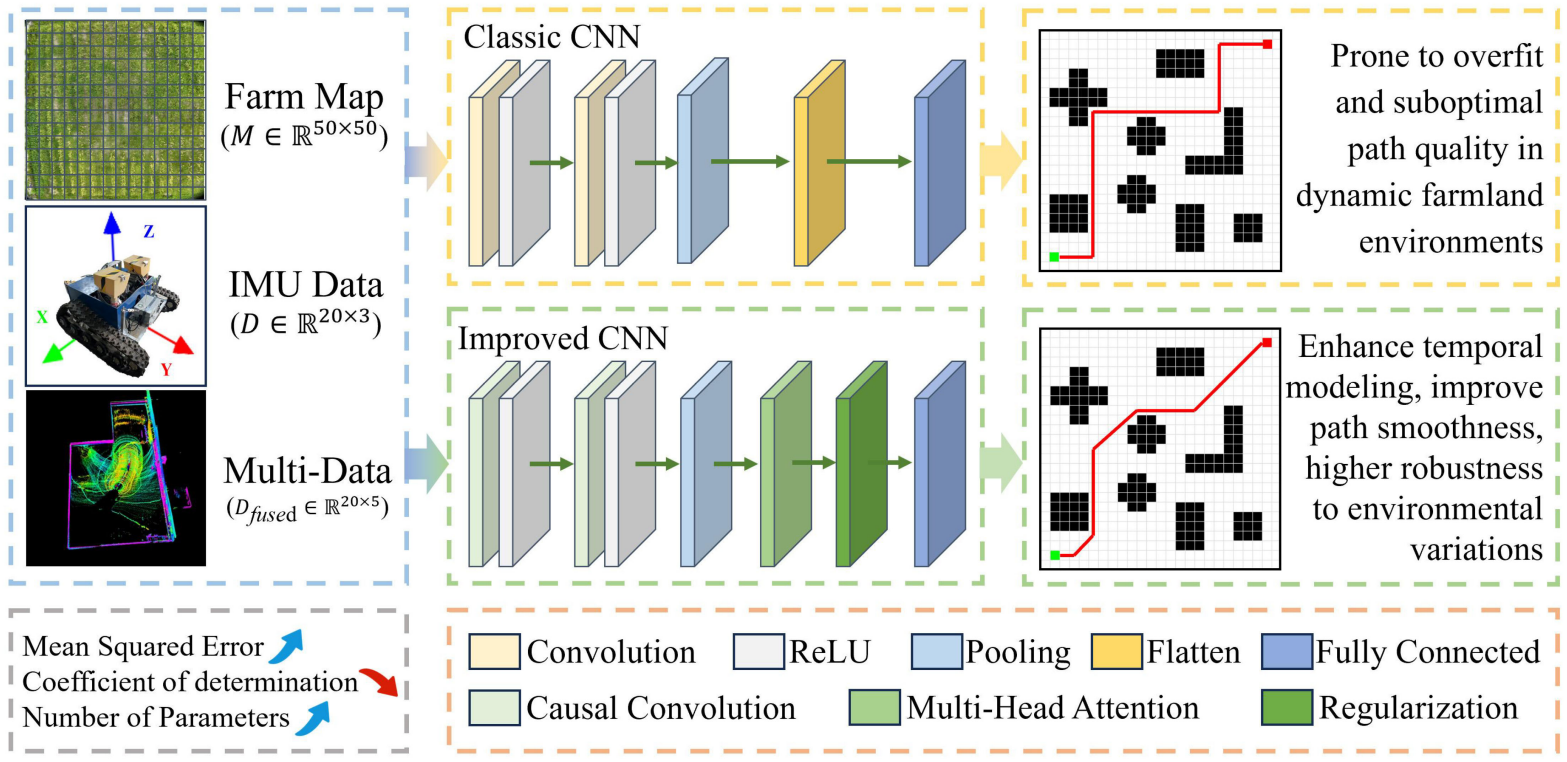
Overall architecture diagram of the improved convolutional neural network module.

The improved CNN module is designed to predict short-term trajectories for agricultural robots operating in complex maize field environments. This module addresses the limitations of conventional CNNs in small-sample, dynamic scenarios—namely, their susceptibility to overfitting and limited adaptability to temporal changes in multimodal data (Zhao et al., 2024b). The overall process includes multimodal data preprocessing, causal convolution-based feature extraction, self-attention-based temporal focus, dimensionality reduction via pooling, and final trajectory prediction. A Gaussian perturbation is applied to the predicted output to generate an initial trajectory for downstream path fusion (**Figure 3**).

Module inputs:

1. Inertial measurement unit (IMU) data $D_{IMU}\in {\mathbb{R}}^{20\times 3}$, including linear acceleration $\left(a_{x},a_{y}\right)$ and angular velocity $\left(\omega_{z}\right)$, sampled at 10 Hz.
2. Fused multimodal data $D_{fused}\in {\mathbb{R}}^{20\times 5}$, integrating GPS position (x,y), Normalized Difference Vegetation Index (NDVI), and environmental states.
3. Map data $M\in {\mathbb{R}}^{50\times 50}$, with a resolution of 0.5 m, containing static obstacle information.

To enhance the model’s sensitivity to dynamic temporal features, input data undergo time synchronization and Kalman filtering. Low-frequency NDVI and GPS signals are interpolated to 10 Hz and aligned with the IMU data to form a temporally unified multimodal input. NDVI data are sourced from drone-acquired hyperspectral images (DJI Mavic 3 Pro, 20 MP resolution, sampled at 1 Hz during flights at 10-m altitude), while GPS data come from robot-mounted receivers (u-blox NEO-M8, 1 Hz). To align with IMU’s 10-Hz sampling, low-frequency signals are interpolated using the cubic spline method (MATLAB’s ‘spline’ function), chosen for smoothness in dynamic fields. Interpolation error analysis shows mean absolute error <0.05 m for GPS positions and <0.02 for NDVI values, minimally impacting prediction accuracy (validated via cross-validation on 100 samples).

The convolution module adopts a two-layer causal convolution structure. The first layer contains 16 convolutional filters and the second 32, each with a kernel size of 3×1, and employs the Rectified Linear Unit (ReLU) activation function. The convolution operation is defined as Equation 2:

(2) $$y_{t}=max(0,\sum_{k=0}^{K}\;w_{k}\cdot x_{t-k})$$

where K=3 and $w_{k}$ denotes the convolution kernel weights. Causal convolution ensures that the current output depends solely on the current and past inputs, which is crucial for modeling the short-term dynamics of IMU signals.

To emphasize key moments—such as turns or imminent obstacle encounters—a multi-head self-attention mechanism is introduced, with four attention heads each of dimension 16. The attention is computed as Equation 3:

(3) $$Attention(Q,K,V)=softmax(\frac{QK^{⊤}}{\sqrt{d_{k}}})V$$

where *Q*, *K*, and *V* represent the query, key, and value matrices, respectively, and $d_{k}=16$ is the scaling factor. This mechanism assigns weights to salient features, enhancing the model’s responsiveness to critical temporal signals.

After feature extraction, max pooling is applied for dimensionality reduction (window size, 2×1; stride, 2), followed by a fully connected layer that outputs the predicted short-term position (x,y). To mitigate overfitting, dropout with a rate of 0.3 and L2 regularization are employed. The loss function is defined as Equation 4:

(4) $$F_{total}=\frac{1}{N}\sum_{i=1}^{N}{\;(y_{i}-{\hat{y}}_{i})}^{2}+\lambda \sum_{j}w_{j}^{2}$$

where λ=0.01; $y_{i}$ and ${\hat{y}}_{i}$ are the ground truth and predicted values, respectively; and $w_{j}$ denotes the model weights.

To enhance the diversity of initial solutions for subsequent path optimization, a Gaussian perturbation is added to the prediction as Equation 5:

(5) $$X_{init}^{t}=X_{CNN}^{t}+N(0,\sigma^{2}),\;\sigma =0.01$$

Moreover, key CNN hyperparameters—such as learning rate and number of attention heads—are co-optimized by the IWOA, with the update rule given by Equation 6:

(6) $$\theta_{i}^{\left(t+1\right)}=\theta_{i}^{\left(t\right)}+2\cdot \text{cos}\;(\frac{\pi t}{2T})\cdot e^{-t/T}\cdot \delta_{i}$$

where $\theta_{i}$ is the *i*th hyperparameter, $\delta_{i}$ is a perturbation term, and *T* is the maximum number of iterations.

#### 2.2.2 Improved A* algorithm module

**Figure 4:**
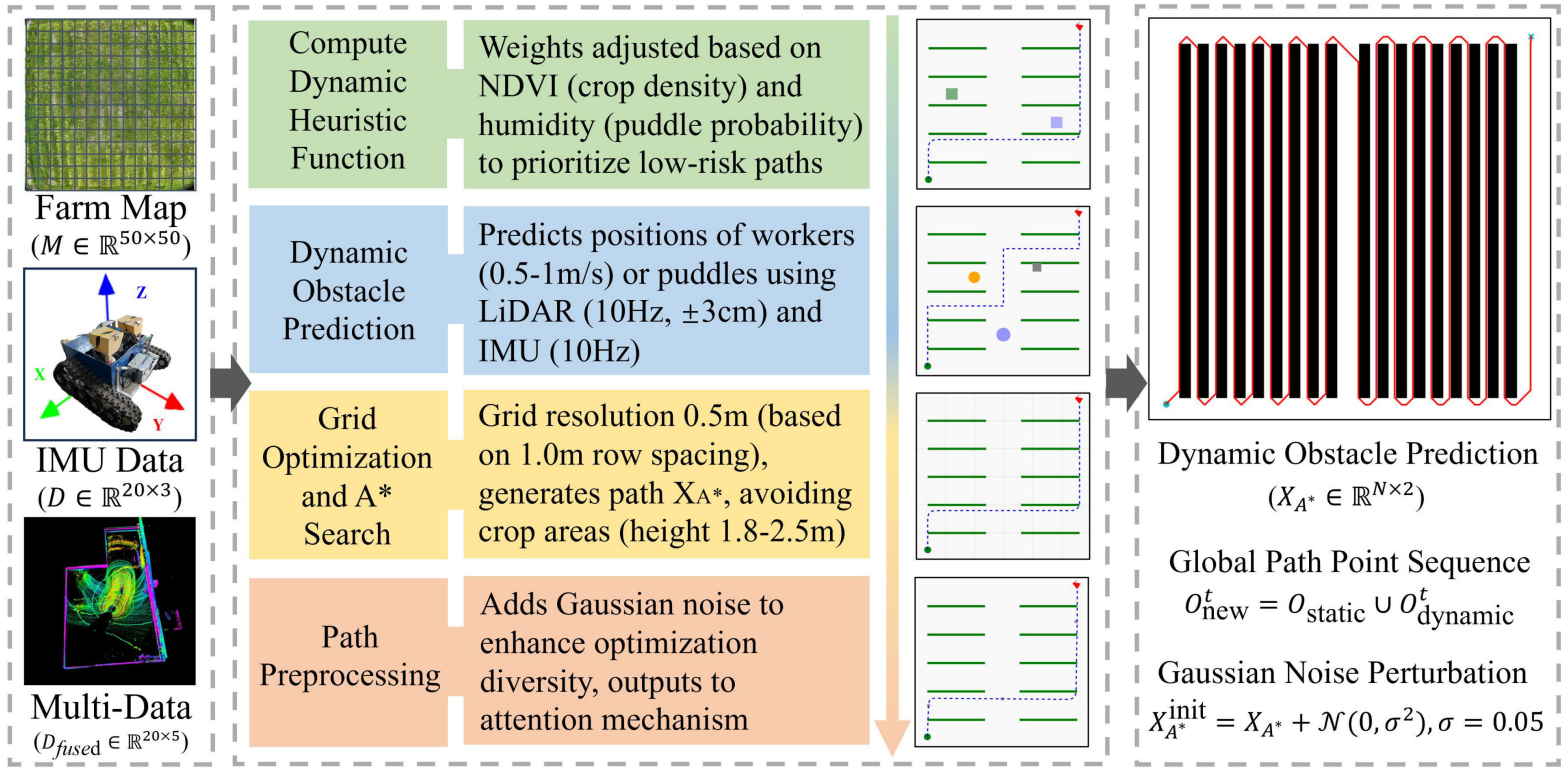
Overall architecture diagram of the improved A* algorithm module.

To satisfy the requirements of global path planning for agricultural robots operating in complex farmland environments, we propose an improved A* algorithm module incorporating environmental perception (**Figure 4**). The module receives two key inputs: a grid-based map $M\in {\mathbb{R}}^{50\times 50}$, with a resolution of 0.5 m representing a 50 m × 50 m maize field, and a multimodal perception matrix $D_{multi}\in {\mathbb{R}}^{20\times 5}$, which fuses LiDAR point clouds, filtered for low-reflectance areas (<10% reflectivity). Camera imagery, analyzed for water signatures using HSV color thresholding, is temporally aligned (10 Hz) with NDVI data. The output is a global waypoint sequence $X_{A^{*}}\in {\mathbb{R}}^{N\times 2}$, which is passed to subsequent optimization modules.

Conventional A* algorithms often rely on Manhattan distance as a heuristic, which is suitable for static environments but proves inadequate in dynamic farmlands featuring mobile obstacles, water patches, and other unstructured elements (Sang et al., 2023). To address this, we introduce a dynamic obstacle-aware heuristic as Equation 7:

(7) h(n)=α·d(n,g)+β·ObstacleRisk(n)

where d(n,g) denotes the Manhattan distance between node *n* and the goal *g*, and ObstacleRisk(n) quantifies the local risk based on NDVI and moisture indicators. The initial parameter weights are set as α=0.7 and β=0.3, with subsequent dynamic tuning within the interval [0.5,1.0] by the IWOA.

To improve responsiveness to dynamic obstacles, the algorithm incorporates a prediction mechanism based on historical state estimation. LiDAR captures point cloud data at 10 Hz, which—along with IMU-derived accelerations $\left(a_{x},a_{y}\right)$ and angular velocity $\omega_{z}$—is processed through Kalman filtering to forecast the positions of moving obstacles over a 5-second horizon. The updated obstacle set is expressed as Equation 8:

(8) $$O_{new}^{t}=O_{static}\cup O_{dynamic}^{t}$$

where $O_{static}$ represents static obstacles and $O_{dynamic}^{t}$ denotes the predicted positions of dynamic obstacles. This enables proactive rerouting and enhances node selection during planning.

In terms of map discretization, the grid resolution is chosen as 0.5 m, aligned with the row spacing and operational lane width in maize fields, to reduce the generation of infeasible nodes within crop zones. To promote solution diversity and enhance global search capabilities in downstream optimization, Gaussian perturbation is applied to the resulting path as Equation 9:

(9) $$X_{A^{*}}^{init}=X_{A^{*}}+N(0,\sigma^{2}),\;\sigma =0.05$$

This step introduces controlled stochasticity while ensuring that all path points lie within the feasible domain Ω, thereby avoiding incursion into crop rows or obstacle regions.

#### 2.2.3 Path fusion module

This module integrates the short-term trajectory $X_{CNN}^{t}\in {\mathbb{R}}^{2}$, generated by the improved CNN, with the global path $X_{A^{*}}\in {\mathbb{R}}^{N\times 2}$, derived from the enhanced A* algorithm. The goal is to produce a high-quality initial path $X_{init}\in {\mathbb{R}}^{N\times 2}$ that adapts to the complex and dynamic conditions of maize field operations and serves as a robust basis for subsequent optimization by the IWOA.

Traditional fusion techniques typically employ static weighted averaging, which lacks adaptability to changing environmental conditions and often leads to suboptimal path alignment. To overcome this, we develop a dynamic fusion strategy based on multi-head self-attention, allowing the fusion weights to be adaptively regulated in response to environmental variations.

First, the local trajectory and global path are concatenated and linearly projected into query (*Q*), key (*K*), and value (*V*) representations as Equation 10:

(10) $$Q=W_{q}[X_{CNN}^{t};X_{A^{*}}],\;K=W_{k}[X_{CNN}^{t};X_{A^{*}}],\;V=W_{v}[X_{CNN}^{t};X_{A^{*}}]$$

where $W_{q},W_{k},\text{and}W_{v}\in {\mathbb{R}}^{d\times 4}$ are trainable weight matrices, and the feature dimension is set to d=16.

Next, four attention heads (each of dimension 16) are used in parallel to compute the attention distribution as Equation 3, with $d_{k}=16$ as the scaling factor.

To further improve adaptability to dynamic conditions, we introduce an environmental context vector $E_{context}\in {\mathbb{R}}^{20}$, extracted from multimodal sensor data $D_{fused}$, and embed it into the attention mechanism as Equation 11:

(11) $$Attention_{contex\text{t}}=softmax(\frac{(Q+W_{e}E_{context})K^{⊤}}{\sqrt{d_{k}}})V$$

where $W_{e}\in {\mathbb{R}}^{d\times 20}$ is the embedding weight matrix.

The fused result is then passed through a fully connected layer to generate the initial trajectory $X_{fused}$, followed by Gaussian noise injection as Equation 12:

(12) $$X_{init}=X_{fused}+N(0,\sigma^{2}),\;\sigma =0.05$$

This stochastic enhancement improves solution diversity and reduces the likelihood of entrapment in local optima, providing a richer solution pool for IWOA-based optimization.

Given the computational limitations of the Jetson Nano embedded platform, we employ a parameter compression strategy to restrict the total number of parameters to approximately 200. Furthermore, we introduce a pre-computation mechanism for attention weights, and we measure latency figures on Jetson Nano (single-threaded, CPU load <50%) over 100 runs using Python’s time module, with mean ± std: attention precompute 0.018 ± 0.002 seconds, total fusion <0.02 seconds, meeting the real-time navigation requirements of field-deployed agricultural robots.

The final fused path $X_{init}$ ensures that all waypoints $(x_{i},y_{i})\in \text{Ω}$, where the feasible domain Ω is defined based on the 1.0 m maize row spacing. Moreover, the path is designed to prioritize the coverage of healthy vegetation regions indicated by NDVI data, achieving greater than 95% coverage for spraying and inspection tasks.

#### 2.2.4 Improved whale optimization algorithm module

**Figure 5:**
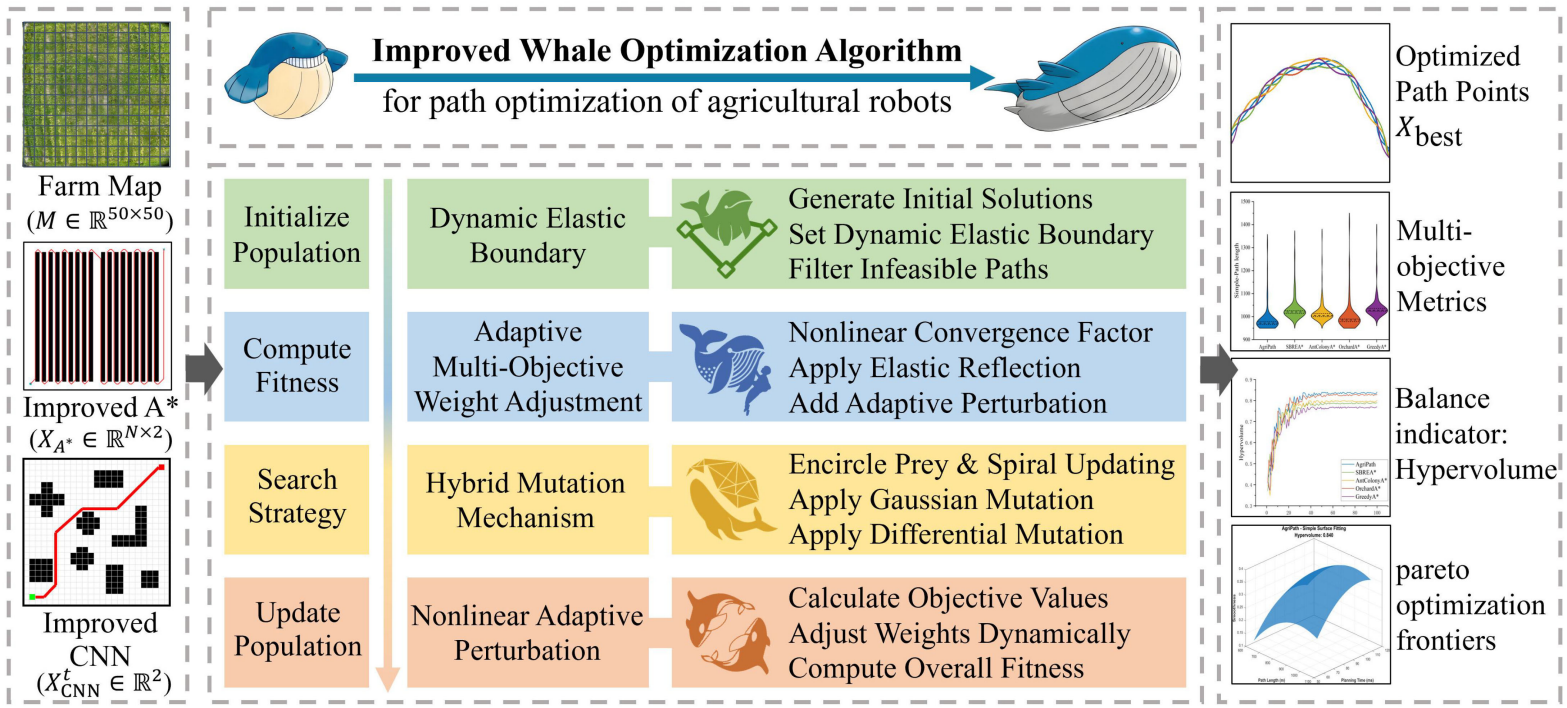
Overall architecture diagram of the improved whale optimization algorithm module.

The IWOA module is designed to achieve multi-objective path optimization in complex maize field environments, targeting path length, smoothness, and planning time (**Figure 5**). The conventional whale optimization algorithm (WOA) simulates the hunting behavior of humpback whales to iteratively search the global solution space and generate an optimal path $X_{best}$. This demonstrates its effectiveness in global optimization problems (Shen et al., 2023). However, in agricultural scenarios, WOA suffers from slow convergence, susceptibility to local optima, and limited adaptability—particularly due to its linear convergence factor and fixed weights, which restrict dynamic multi-objective trade-offs (Yuan et al., 2025). To address these limitations, the proposed IWOA operates synergistically with the improved CNN, improved A* algorithm, and path fusion modules, forming a closed-loop system that combines bottom-up path prediction with top-down global optimization, thereby significantly enhancing algorithmic performance.

Initially, the IWOA introduces a dynamic elastic boundary constraint to prevent path points from exceeding permissible limits. The boundary update is defined as Equation 13:

(13) $$\begin{array}{c} X_{min}^{t}=X_{min}^{0}+0.5(1-\frac{t}{T})(X_{max}^{0}-X_{min}^{0})\\ X_{max}^{t}=X_{max}^{0}-0.5(1-\frac{t}{T})(X_{max}^{0}-X_{min}^{0}) \end{array}$$

where $X_{min}^{0}$ and $X_{max}^{0}$ are the initial boundaries, respectively, and *t* and *T* represent the current and maximum iteration counts, respectively. These boundaries dynamically contract toward the feasible farmland region as iterations progress. An elastic reflection strategy is employed as Equation 14:

(14) $$X_{i}^{\left(t+1\right)}=X_{min}^{t}+0.1(1-\frac{t}{T})(X_{max}^{t}-X_{min}^{t})$$

This approach retains useful information from out-of-bound solutions, enriching search diversity and reducing ineffective iterations.

Second, a non-linear adaptive convergence factor enhances search capability across different optimization stages as Equation 15:

(15) $$\begin{array}{c} a(t)=2\cdot \text{cos}(\frac{\pi t}{2T})\cdot e^{-t/T} \end{array}$$

This factor encourages broad exploration early on to increase path diversity while gradually focusing on local refinement in later stages. Additionally, a fitness-based perturbation term is introduced to improve escape from local optima as Equation 16:

(16) $$\delta_{i}=\frac{f(X_{i}^{t})-f_{min}}{f_{max}-f_{min}}\cdot rand(0,1)$$

The position update rule is modified as Equation 17:

(17) $$X_{i}^{\left(t+1\right)}=X_{rand}^{t}-A\cdot |C\cdot X_{rand}^{t}-X_{i}^{t}|+\delta_{i}$$

where *A* and *C* are the original WOA coefficients, and rand(0,1) is a random number in the interval [0, 1]. This modification improves adaptability to path quality and obstacle avoidance.

To boost global search efficacy, the IWOA integrates Gaussian perturbation and differential evolution (DE) mechanisms. The Gaussian perturbation is expressed as Equation 18:

(18) $$X_{i}^{\left(t+1\right)}=X_{i}^{t}+N(0,\sigma ),\;\sigma =0.1\cdot e^{-t/T}$$

The DE strategy is given by Equation 19:

(19) $$X_{i}^{\left(t+1\right)}=X_{i}^{t}+0.5(X_{r1}^{t}-X_{r2}^{t})$$

These hybrid strategies are probabilistically applied to subsets of the population, effectively maintaining diversity and mitigating premature convergence.

Furthermore, the IWOA implements an adaptive weight adjustment scheme to dynamically balance the three objectives of path length, smoothness, and planning time. The weight for each objective is updated based on the fitness of the current best solution, as in Equation 20:

(20) $$w_{i}(t)=w_{i}^{0}(1+0.2\cdot \frac{f_{i}(X_{best}^{t})}{f_{i,max}})$$

where $f_{i}$ denotes the normalized objective values for path length, smoothness, and planning time; $f_{i,max}$ represents the maximum value of each objective; and the initial weights $w_{1}^{0},w_{2}^{0},\text{and}w_{3}^{0}$ are set to 0.4, 0.3, and 0.3, respectively.

The overall multi-objective optimization function is formulated as Equation 21:

(21) $$\begin{array}{c} F(X)=w_{1}(t)\cdot \frac{f_{1}}{f_{1,max}}+w_{2}(t)\cdot \frac{f_{2}}{f_{2,max}}+w_{3}(t)\cdot \frac{f_{3}}{f_{3,max}} \end{array}$$

This comprehensive approach enables the efficient and balanced optimization of critical path planning metrics within the challenging operational context of maize fields.

#### 2.2.5 Improved Douglas–Peucker algorithm module

**Figure 6:**
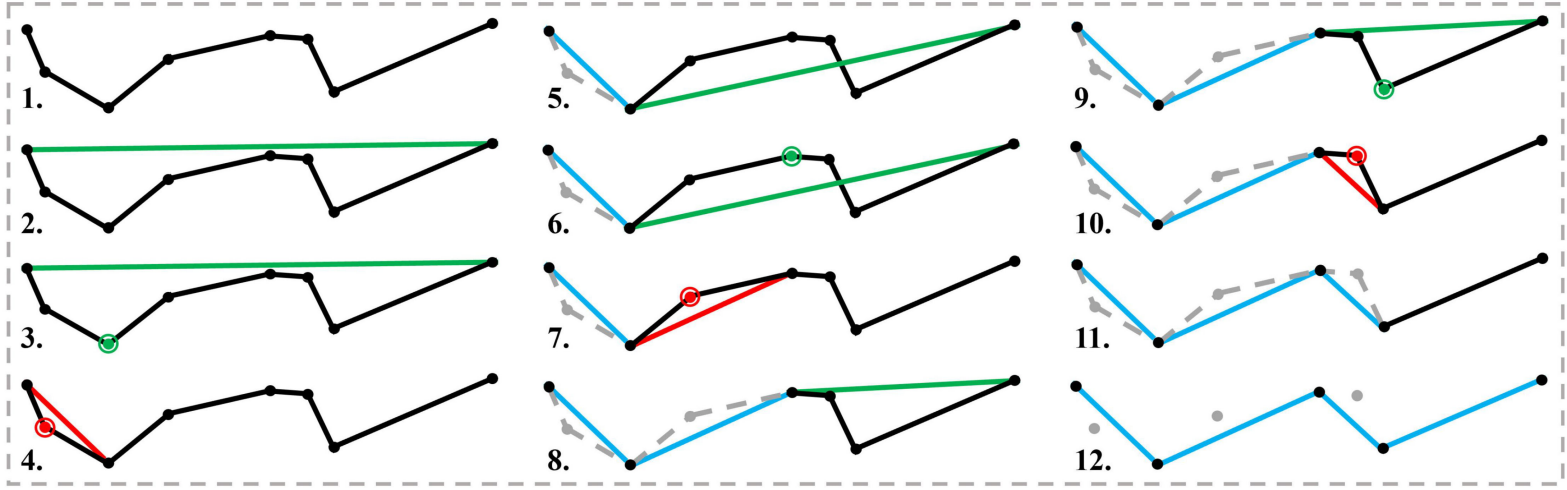
Improved Douglas–Peucker algorithm module optimization process diagram.

The improved Douglas–Peucker algorithm module introduces a dynamic adaptive thresholding mechanism that incorporates environmental features, alongside a cubic B-spline smoothing strategy to improve path quality (**Figure 6**). This module performs path simplification and smoothing on the global path $X_{\text{best}}\in {\mathbb{R}}^{N\times 2}$ generated by the IWOA, aiming to reduce the number of waypoints, enhance trajectory smoothness, and decrease computational complexity, which is essential for real-time navigation in dense crop environments (Manrique-Cordoba et al., 2025). It operates synergistically with the improved CNN, improved A* algorithm, path fusion, and the IWOA modules to form a closed-loop path planning system integrating local perception, global planning, path fusion, multi-objective optimization, and smoothing, thus fulfilling the real-time constraints of embedded systems in complex agricultural environments.

The module inputs include the IWOA output path $X_{\text{best}}$, multimodal sensor fusion data $D_{fused}\in {\mathbb{R}}^{20\times 5}$, and map information $M\in {\mathbb{R}}^{50\times 50}$. The output is the simplified and smoothed path $X_{simplified}\in {\mathbb{R}}^{M\times 2}$, where M<N, intended for the navigation command integration module.

Path simplification employs a recursive segmentation algorithm that calculates the perpendicular distance of path points to the line segment connecting the segment’s endpoints. The threshold $\epsilon_{t}$ dynamically adapts according to environmental conditions as Equation 22:

(22) $$\epsilon_{t}=\epsilon_{0}\cdot (1+0.2\times Humidity+0.3\times NDVI)$$

where the initial threshold $\epsilon_{0}=0.1\text{m}$, and both Humidity and NDVI reflect local moisture and crop density, respectively. This adaptive thresholding ensures the preservation of critical path features while enabling environment-aware simplification.

Following simplification, the path is smoothed using cubic B-spline interpolation as Equation 23:

(23) $$P\left(t\right)=\sum_{i=0}^{M}B_{i,3}\left(t\right)\cdot (x_{i},y_{i}),\;t\in [0,1]$$

where $B_{i,3}(t)$ denotes the cubic B-spline basis functions and $\left(x_{i},y_{i}\right)$ are the coordinates of control points. This method significantly improves continuity and curvature smoothness, ensuring stable traversal within the 1.0-m maize row spacing.

To address dynamic obstacles such as water puddles, the module integrates real-time detection using LiDAR point clouds combined with NDVI data to delineate obstacle regions $O_{new}$. When a path point $(x_{i},y_{i})\in O_{new}$ is detected, local path reconstruction is triggered, with the optimization objective as Equation 24:

(24) $$X_{simplified}^{'}=arg\;\underset{X}{min}\sum_{i}\;d(X_{i},X_{best}),\;s.t.(x_{i},y_{i})\notin O_{new}$$

where d(·) denotes the Euclidean distance between points, ensuring that the revised path avoids newly identified obstacles.

For compatibility with embedded computing constraints, the module precomputes the B-spline basis function matrix to reduce online computational overhead. The parameter count is limited to approximately 100, and the combined simplification and smoothing latency is controlled below 0.02 seconds, satisfying the real-time processing requirement of under 0.1 seconds per path point.

The final output path $X_{simplified}$ guarantees that all waypoints reside within the feasible domain Ω defined by the 1.0-m row spacing and preferentially covers healthy crop regions indicated by NDVI. This provides a reliable, efficient trajectory foundation for the agricultural robot’s subsequent spraying and inspection tasks.

#### 2.2.6 Navigation command integration module

**Figure 7:**
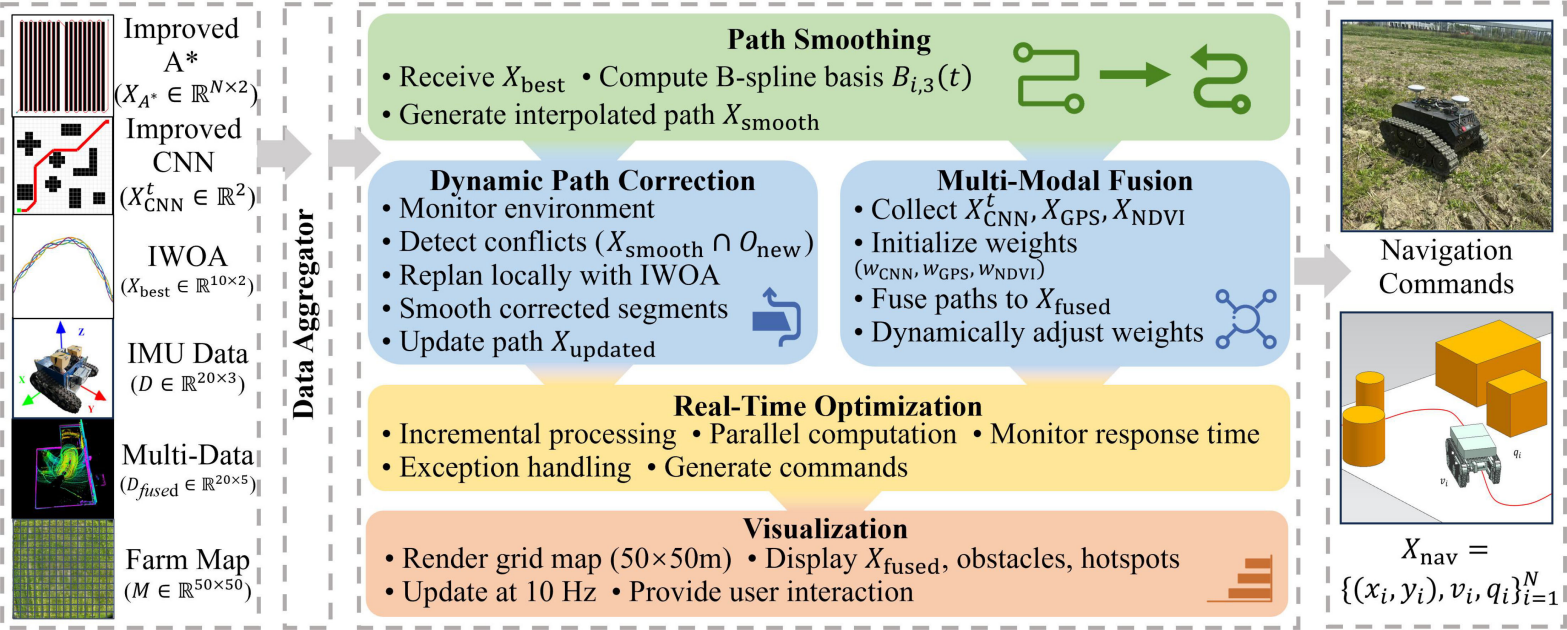
Overall architecture diagram of the navigation command integration module.

The navigation command integration module serves as the final stage in the path planning and optimization pipeline (**Figure 7**). It consolidates the global path $X_{best}\in {\mathbb{R}}^{N\times 2}$ produced by the IWOA, the simplified path $X_{simplified}\in {\mathbb{R}}^{M\times 2}$ generated via the improved Douglas–Peucker algorithm, the short-term trajectory prediction $X_{CNN}^{t}\in {\mathbb{R}}^{2}$ from the improved CNN, the global path ${\hat{X}}_{A}\in {\mathbb{R}}^{N\times 2}$ from the improved A* algorithm, multimodal sensor fusion data $D_{fused}\in {\mathbb{R}}^{20\times 5}$, and map data $M\in {\mathbb{R}}^{50\times 50}$. This integration addresses the limitations of conventional navigation methods that struggle to fuse multi-source path information and lack dynamic adaptability, often resulting in path deviations and collisions under dynamic environmental conditions such as rainfall.

The module generates a set of executable navigation commands for the agricultural robot as Equation 25:

(25) $$X_{nav}=\{(x_{i},y_{i},v_{i},q_{i})i=1,\ldots ,M\}$$

where $\left(x_{i},y_{i}\right)$ denote the coordinates of each waypoint; $v_{i}\in [0.5,2]\text{ m}/\text{s}$ represents the travel velocity; and $q_{i}\in [0.1,1]\text{ L}/{\text{m}}^{2}$ specifies the spraying operation parameters. The module aims to enhance path smoothness, environmental adaptability, and operational efficiency to meet the complex demands of maize field tasks.

A dynamic weighted fusion strategy centered on the simplified path $X_{simplified}$ is proposed, incorporating the short-term predicted trajectory $X_{CNN}^{t}$ and the global path ${\hat{X}}_{A}$ to produce a comprehensive fused path as Equation 26:

(26) $$X_{fused}=w_{DP}\cdot X_{simplified}+w_{CNN}\cdot X_{CNN}^{t}+w_{A}\cdot {\hat{X}}_{A}$$

with initial weights set as $w_{DP}=0.5$, $w_{CNN}=0.3$, and $w_{A}=0.2$. These weights are dynamically adjusted within the range [0.1, 0.7] by the IWOA based on adaptive optimization informed by NDVI and humidity sensor data, thereby enhancing the accuracy and robustness of path fusion.

To tackle dynamic obstacles, the module implements a real-time path correction mechanism. Leveraging LiDAR point clouds (10 Hz, ± 3 cm accuracy) and IMU data (10 Hz, including accelerations $a_{x},a_{y}$ and angular velocity $\omega_{z}$), newly detected obstacle regions $O_{\text{new}}$ are identified. If any path point $(x_{i},y_{i})\in O_{new}$, a local path update is triggered as Equation 27:

(27) $$X_{fused}^{'}=arg\underset{X}{\;min}\sum_{i}\;d(X_{i},X_{fused})\;s.t.(x_{i},y_{i})\notin O_{new}$$

where d(·) denotes the distance metric between path points. This local adjustment is performed via the IWOA over 10 short iterations, ensuring that an obstacle-avoiding path is generated within 0.05 seconds, significantly improving real-time responsiveness and collision avoidance success rates.

To accommodate the computational constraints of the Jetson Nano embedded platform, the module employs incremental weight updating and precomputed matrix strategies. Per-point latency (<0.03 seconds) is averaged over 50 trials under multi-threaded execution (Robot Operating System ROS framework), with system load 60%–80%, including sensor fusion, and std deviation of 0.005 seconds, ensuring 10-Hz compatibility. The parameter count is limited to approximately 150, and per-point navigation computation latency is maintained below 0.03 seconds, satisfying stringent real-time requirements.

Furthermore, the module integrates real-time visualization capabilities, dynamically rendering path points, obstacles, and operational status on a 50 m × 50 m grid map (0.5-m resolution) at 10 Hz. This feature facilitates continuous monitoring and strategic adjustment by operators.

## 3 Experiments and results

To validate the performance of the proposed path planning and optimization framework for agricultural robots operating within complex field environments, this chapter presents a series of representative experimental scenarios. Comprehensive evaluations are conducted across multiple dimensions, including path length, planning time, smoothness, energy consumption control, and obstacle avoidance capability. Furthermore, ablation studies analyze the contribution of key modules to overall system performance.

### 3.1 Experimental setup

**Figure 8:**
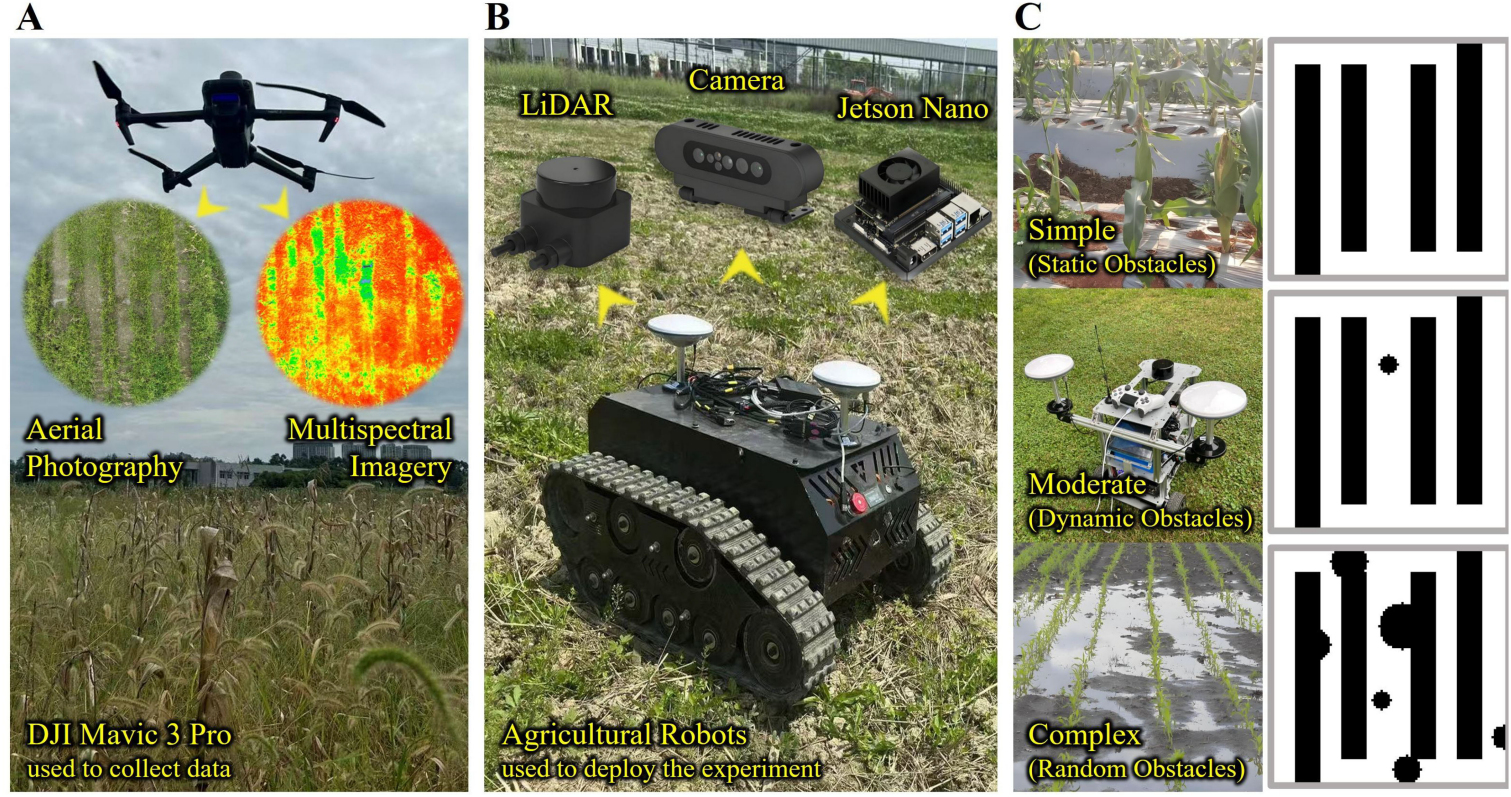
The experimental environment and equipment. **(A)** DJI Mavic 3 Pro used to collect data. **(B)** Agricultural robots used to deploy the experiment. **(C)** Three experimental environments.

To assess the practicality and robustness of the path planning approach in complex farmland conditions, experiments are conducted within a 50 m × 50 m subsection of the demonstration site. The experimental platform comprises a DJI Mavic 3 Pro drone, equipped with a 20-MP camera and a hyperspectral sensor, and an unmanned agricultural robot, forming a comprehensive sensing and navigation system (**Figures 8A,B**). The robot, custom-built with dimensions of 1.2 m × 0.8 m × 0.6 m, is fitted with a LeiShen 16-line LiDAR, RGB camera, and an IMU (MPU-6050), optimized to minimize occlusion within the 1.0-m row spacing of the maize field. This platform, based on the Jetson Nano embedded system, enables real-time perception and processing of multi-source sensory data, with sensor configurations ensuring 10-Hz data fusion.

To enhance the algorithm’s adaptability to real-world agricultural complexities, three categories of representative obstacles are introduced into the experimental scenarios (**Figure 8C**):

1. Static obstacles (simple): including crops and field ridges, occupying approximately 40% of the area, with heights ranging from 0.3 to 1.5 m, fixed in position;
2. Dynamic obstacles (moderate): comprising other agricultural robots and field workers, moving at speeds between 0.5 and 1.0 m/s, exhibiting spatiotemporal randomness; and
3. Random obstacles (complex): water puddles formed after rainfall, with diameters ranging from 0.5 to 5.0 m and depths between 0.1 and 0.3 m, characterized by uncertainty and variability.

Given the maize row spacing of merely 1.0 m, coupled with dense vegetation and substantial occlusion, the agricultural robot faces stringent constraints on path accessibility and demands high-precision obstacle avoidance. The experimental region is finely modeled using a regular grid with 0.5-m resolution to support high-accuracy path planning and analysis.

### 3.2 Hyperparameter configuration

To enhance the operational efficiency and responsiveness of each algorithmic module, core submodule hyperparameters are systematically tuned through grid search combined with cross-validation. The detailed configurations are summarized in **Table 2**.

**Table 2:** Hyperparameter configuration of each module.

Module	Parameter name	Value
Improved CNN	Input size	64 × 64
	Optimizer	Adam
	Learning rate	0.0005
	Batch size	32
	Max epochs	100
Improved A* algorithm	Grid resolution	0.5 m
	Heuristic weight factor α	1.2
	Heuristic strategy	Manhattan distance first
Path fusion	Channel attention compression ratio r	8
	Spatial attention kernel size	7 × 7
	Fusion weight factor β	0.5
IWOA	Initial population size	30
	Max iterations	100
	Convergence factor a	Linearly decreases from 2 to 0
	Multi-objective weights (path length, energy, smoothness, and obstacle avoidance)	0.4, 0.3, 0.2, 0.1
Improved Douglas–Peucker algorithm	Distance threshold ϵ	0.5 m
Navigation command decoding module	Control frequency	10 Hz
	Instruction buffer window length	10
	Path point refresh threshold	1.0 m

CNN, convolutional neural network; IWOA, improved whale optimization algorithm.

This hyperparameter scheme has been refined through multiple simulation cycles and field trials, demonstrating superior convergence speed and navigation stability. It is well-suited for generating paths and performing real-time obstacle avoidance control within dynamically complex agricultural environments.

### 3.3 Results

#### 3.3.1 Path planning performance of algorithms

**Figure 9:**
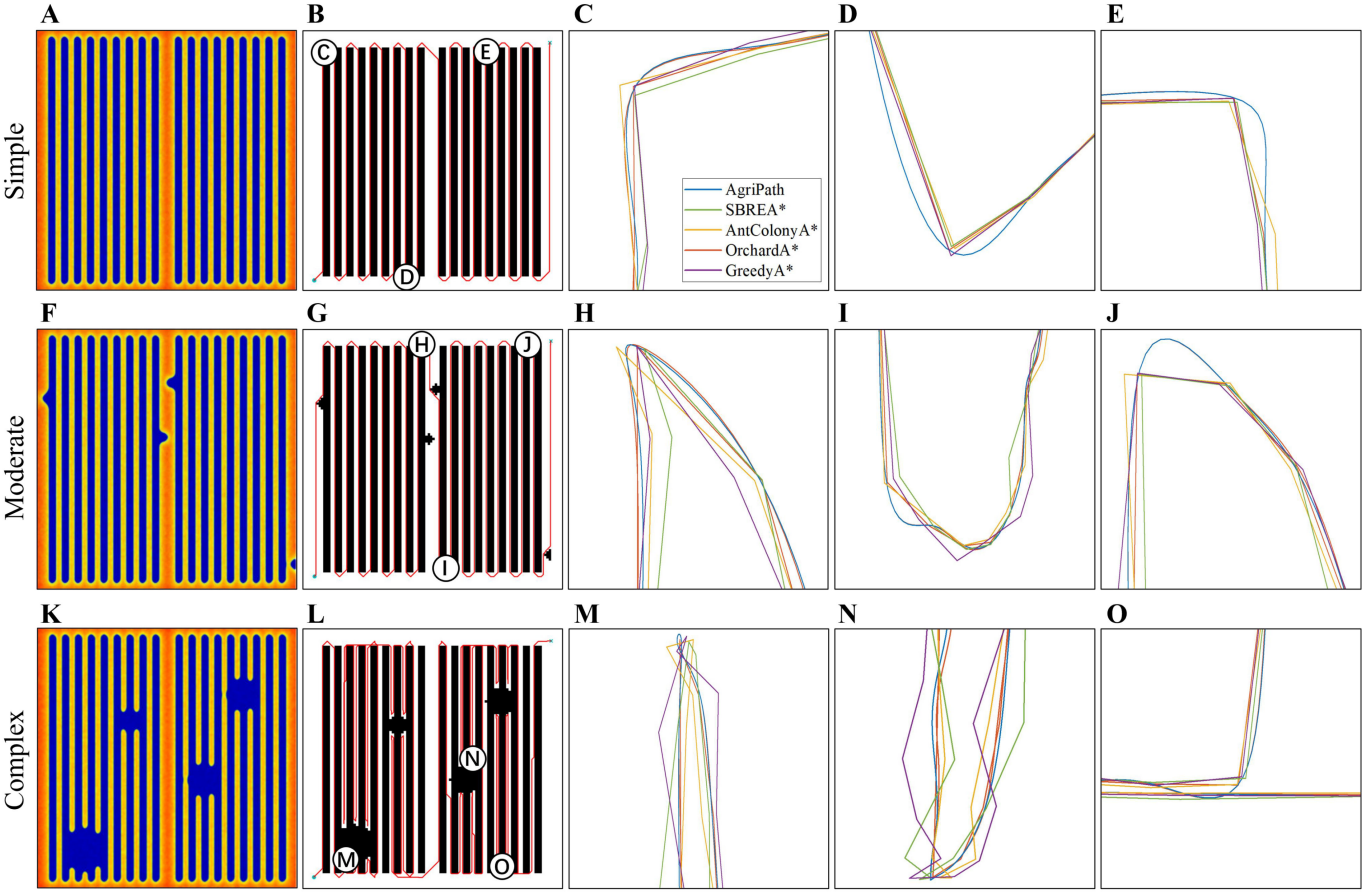
Path planning performance of algorithm results. **(A)** Heat map of the feasible domain of the agricultural robot in the simple scenario. **(B)** Schematic diagram of path planning in the simple scenario. **(C)** Schematic diagram of path planning for point C in the simple scenario, zoomed in to 1 m * 1 m. **(D)** Schematic diagram of path planning for point D in the simple scenario, zoomed in to 1 m * 1 m. **(E)** Schematic diagram of path planning for point E in the simple scenario, zoomed in to 1 m * 1 m. **(F)** Heat map of the feasible domain of the agricultural robot in the moderate scenario. **(G)** Schematic diagram of path planning in the moderate scenario. **(H)** Schematic diagram of path planning for point H in the moderate scenario, zoomed in to 1 m * 1 m. **(I)** Schematic diagram of path planning for point I in the moderate scenario, zoomed in to 1 m * 1 m. **(J)** Schematic diagram of path planning for point J in the moderate scenario. **(K)** Heat map of the feasible domain of the agricultural robot in the complex scenario. **(L)** Schematic diagram of path planning in the complex scenario. **(M)** Select point M in the complex scenario, zoomed in to a 1 m * 1 m path planning diagram. **(N)** Select point N in the complex scenario, zoomed in to a 1 m * 1 m path planning diagram. **(O)** Select point O in the complex scenario, zoomed in to a 1 m * 1 m path planning diagram.

This section evaluates the performance of the AgriPath in navigating complex maize fields through 100 trials conducted in three representative scenarios: simple, moderate, and complex. The performance is systematically compared against advanced algorithms, including SBREA* (Xu et al., 2024), Ant Colony A* (Dai et al., 2019), Orchard A* (Zhang et al., 2024a), and Greedy A* (Xiang et al., 2022), focusing on path length and smoothness. The shortest path lengths achieved by each algorithm across the three scenarios are presented in **Table 3**. The heat map shows the feasible domain of agricultural robots (**Figures 9A,F,K**). Path smoothness results are derived from nine representative waypoints, with comparative analysis conducted via magnified path trajectory visualizations (1 m × 1 m) for waypoints (**Figures 9B,G,L**). The “Shortest Path” value of 950.00 m in **Table 3** represents the theoretical minimum path length in an obstacle-free 50 m × 50 m maize field, computed using Dijkstra’s algorithm on a 0.5-m-resolution grid map after upsampling for precision. This serves as a baseline for evaluating algorithmic efficiency in obstacle-laden scenarios, assuming straight-line traversal along row spacing with minimal turns.

**Table 3:** Statistics of the shortest path lengths achieved by each algorithm across the three scenarios.

Algorithm	Simple	Moderate	Complex
Shortest Path	950.00 m	–	–
AgriPath	971.34 m	979.51 m	1,457.32 m
SBREA*	1,018.94 m	1,032.67 m	1,658.38 m
Ant Colony A*	1,003.34 m	1,014.57 m	1,623.95 m
Orchard A*	983.45 m	996.34 m	1,520.32 m
Greedy A*	1,026.34 m	1,041.32 m	1,693.52 m

AgriPath demonstrates exceptional path planning performance across the simple, moderate, and complex scenarios, leveraging multimodal data fusion—integrating LiDAR, NDVI, and RGB imagery—with advanced algorithmic optimizations to generate concise and smooth paths. This performance significantly surpasses that of SBREA*, Ant Colony A*, Orchard A*, and Greedy A*. In the simple scenario, AgriPath achieves a path length of 971.34 m, closely approximating the optimal reference path of 950 m, outperforming the longer paths of competing algorithms ranging from 1,018.94 to 1,026.34 m. Notably, SBREA*’s path of 1,018.94 m is protracted due to its lack of dynamic adaptability, whereas AgriPath’s improved A* algorithm, incorporating a dynamic weighting mechanism, adeptly navigates inter-row obstacles in maize fields, yielding compact and smooth trajectories (**Figures 9C–E**). In the moderate scenario, AgriPath records a path length of 979.51 m, surpassing other algorithms’ paths ranging from 996.34 to 1,041.32 m. For instance, Greedy A*’s 1,041.32-m path struggles with dynamic obstacles due to its simplistic strategy, while AgriPath employs an improved CNN for short-term predictions and an IWOA to dynamically circumvent obstacles such as puddles, resulting in shorter and more adaptable paths (**Figures 9H–J**). In the complex scenario, AgriPath’s path length of 1,457.32 m, although 53% longer than the reference path, is substantially shorter than competitors’ paths, ranging from 1,520.32 to 1,693.52 m. For example, Ant Colony A*’s 1,623.95-m path suffers from slow iterative convergence, whereas AgriPath utilizes the improved Douglas–Peucker algorithm to streamline path points, enhancing smoothness and stability, thus demonstrating robust adaptability. In contrast, SBREA* and Greedy A* produce longer, more convoluted paths, while Ant Colony A* and Orchard A* exhibit zigzagging or abrupt turns in dynamic, high-complexity environments (**Figures 9M–O**).

#### 3.3.2 Stability analysis of algorithm performance

This section assesses the stability of AgriPath compared to SBREA*, Ant Colony A*, Orchard A*, and Greedy A* through 100 path planning trials across simple, moderate, and complex scenarios, focusing on path length, smoothness, and planning time distributions. Smoothness is measured as the mean curvature magnitude along the path $X=\{(x_{1},y_{1}),\ldots ,(x_{N},y_{N})\}$, as in Equation 28:

(28) $$\kappa_{i}=\frac{\left|\frac{y_{i+1}-2y_{i}+y_{i-1}}{{\left(x_{i+1}-x_{i}\right)}^{2}}\right|}{{\left(1+{\left(\frac{y_{i+1}-y_{i-1}}{x_{i+1}-x_{i-1}}\right)}^{2}\right)}^{\frac{3}{2}}},\;S=\frac{1}{N-2}\overset{N-1}{\underset{i=2}{\sum }}\kappa_{i}$$

Lower *S* indicates smoother paths.

Optimal results are summarized in **Table 4**. Violin plots illustrating the distributional characteristics of these metrics, including medians and interquartile ranges, validate AgriPath’s superior performance and stability across key indicators.

**Table 4:** Statistics of the best results of the algorithms in path length, smoothness, and planning time for 100 experiments in simple, moderate, and complex scenarios.

Algorithm	Metric	Simple	Moderate	Complex
AgriPath	Path length (m)	971.34	979.51	1,457.32
	Smoothness	0.4033	0.4125	0.5326
	Planning time (ms)	125.1	141.6	200.3
SBREA*	Path length (m)	1,018.94	1,032.67	1,658.38
	Smoothness	0.4547	0.4663	0.5827
	Planning time (ms)	132.8	147.2	237.6
Ant Colony A*	Path length (m)	1,003.34	1,014.57	1,623.95
	Smoothness	0.4428	0.4554	0.5767
	Planning time (ms)	130.6	145.5	225.2
Orchard A*	Path length (m)	983.45	996.34	1,520.32
	Smoothness	0.4122	0.4254	0.5467
	Planning time (ms)	127.3	142.4	209.2
Greedy A*	Path length (m)	1,026.34	1,041.32	1,693.52
	Smoothness	0.4874	0.4925	0.6027
	Planning time (ms)	134.7	148.3	247.2

**Figure 10:**
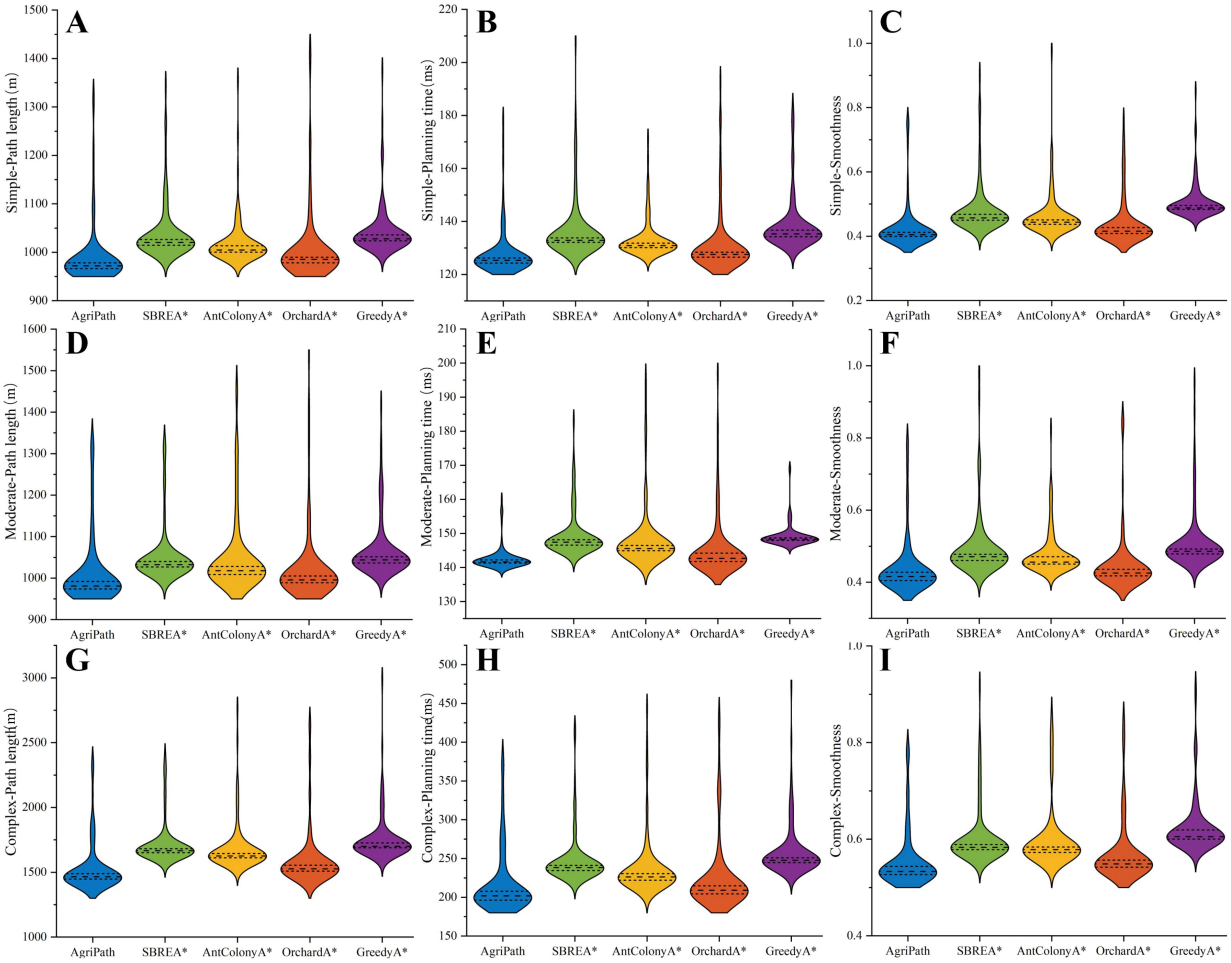
Violin plots of the path length, smoothness, and planning time distributions of all algorithms for 100 experiments in simple, moderate, and complex scenarios. **(A)** Path length distribution of algorithms in simple scenarios. **(B)** Planning time distribution of algorithms in simple scenarios. **(C)** Smoothness distribution of algorithms in simple scenarios. **(D)** Path length distribution of algorithms in moderate scenarios. **(E)** Planning time distribution of algorithms in moderate scenarios. **(F)** Smoothness distribution of algorithms in moderate scenarios. **(G)** Path length distribution of algorithms in complex scenarios. **(H)** Planning time distribution of algorithms in complex scenarios. **(I)** Smoothness distribution of algorithms in complex scenarios.

AgriPath consistently generates paths with shorter lengths, higher smoothness, and reduced planning times across all three scenarios, significantly outperforming SBREA*, Ant Colony A*, Orchard A*, and Greedy A*. In the simple scenario, AgriPath’s path length distribution ranges from 970 to 990 m, with a median of 975 m, closely aligning with the optimal reference path and surpassing other algorithms’ ranges of 1,010 to 1,040 m. For instance, SBREA*’s 1,010–1,030-m range reflects path elongation due to limited dynamic adaptability. AgriPath’s smoothness distribution spans 0.40–0.42, with a median of 0.41, outperforming competitors’ ranges of 0.41–0.50, while its planning time median of 120–130 ms is lower than others’ 125–140 ms, demonstrating efficient computation (**Figures 10A–C**). In the moderate scenario, AgriPath’s path length distribution ranges from 975 to 990 m, with a median of 980 m, outperforming competitors’ 990–1,055-m ranges, such as Greedy A*’s 1,030–1,055 m, which struggles with dynamic obstacles due to its greedy strategy. AgriPath’s smoothness distribution of 0.40–0.43, with a median of 0.41, surpasses others’ 0.42–0.51, and its planning time median of 135–150 ms is lower than competitors’ 138–155 ms, reflecting the efficacy of its improved CNN and the IWOA for dynamic obstacle navigation (**Figures 10D–F**). In the complex scenario, AgriPath’s path length distribution ranges from 1,400 to 1,500 m, with a median of 1,450 m, outperforming competitors’ 1,480–1,750-m ranges, notably Ant Colony A*’s 1,550–1,650 m, which suffers from slow convergence. AgriPath’s smoothness distribution of 0.50–0.55, with a median of 0.52, exceeds others’ 0.53–0.63, and its planning time median of 190–210 ms is lower than competitors’ 200–255 ms, underscoring its robust adaptability to complex environments (**Figures 10G–I**). Optimal results further substantiate AgriPath’s advantages: in the simple scenario, 971.34 m, 0.4033, and 125.1 ms; in the moderate scenario, 979.51 m, 0.4125, and 141.6 ms; and in the complex scenario, 1,457.32 m, 0.5326, and 200.3 ms. They all surpass competing algorithms.

#### 3.3.3 Multi-objective optimization performance analysis

This section evaluates the multi-objective optimization capabilities of AgriPath compared to SBREA*, Ant Colony A*, Orchard A*, and Greedy A* using the hypervolume (HV) indicator, which quantifies the volume of the objective space dominated by the Pareto front relative to a reference point *R*. For a solution set $S=\{s_{1},s_{2},\ldots ,s_{n}\}$ with objectives $f_{1}$ (path length), $f_{2}$ (smoothness), and $f_{3}$ (planning time), the HV is defined as Equation 29:

(29) $$HV(S)=Volume(\underset{s_{i}\epsilon s}{\cup }\left[f_{1}(s_{i}),r_{1}]\times [f_{2}(s_{i}),r_{2}]\times [f_{3}(s_{i}),r_{3}\right])$$

where the reference point is $R=(r_{1},r_{2},r_{3})=(2,000\text{ m},\;1.0,\;500\text{ ms})$, chosen based on the maximum observed values across all scenarios to ensure consistent normalization. The objectives are normalized as: $f_{1}^{'}=\frac{f_{1}}{2000},\;f_{2}^{'}=\frac{f_{2}}{1.0},\text{and }f_{3}^{'}=\frac{f_{3}}{500}$.

Optimal hypervolume values are presented in **Table 5**. Hypervolume convergence curves over 100 iterations, demonstrating AgriPath’s superior convergence stability and multi-objective optimization performance.

**Table 5:** Statistics of the algorithms’ optimal hypervolume values.

Algorithm	Simple	Moderate	Complex
AgriPath	0.84	0.8	0.64
SBREA*	0.79	0.76	0.61
Ant Colony A*	0.8	0.78	0.62
Orchard A*	0.83	0.8	0.63
Greedy A*	0.77	0.75	0.62

**Figure 11:**
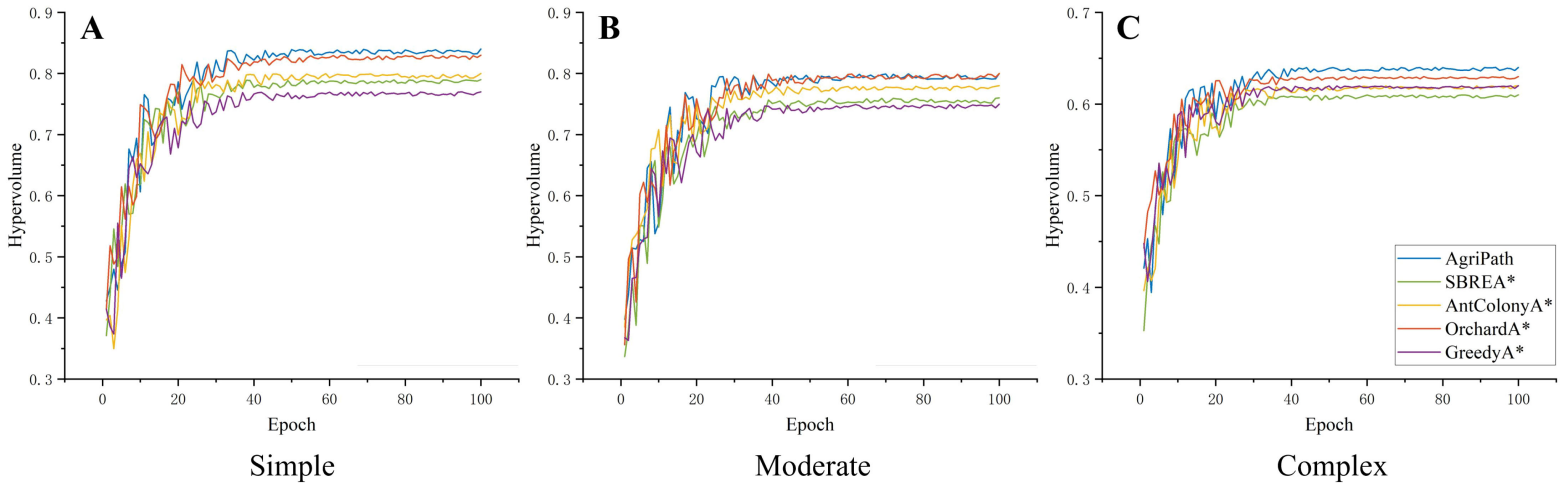
Convergence diagrams of multi-objective optimization performance. **(A)** Hypervolume convergence change diagram of the algorithms in a simple scenario. **(B)** Hypervolume convergence change diagram of the algorithms in a moderate scenario. **(C)** Hypervolume convergence change diagram of the algorithms in a complex scenario.

AgriPath exhibits faster hypervolume growth and greater stability across all three scenarios, significantly outperforming SBREA*, Ant Colony A*, Orchard A*, and Greedy A*. In the simple scenario, AgriPath’s hypervolume rapidly increases from 0.4 to 0.84, stabilizing after just 60 iterations, surpassing competitors’ ranges of 0.77 to 0.83. For instance, SBREA* reaches only 0.79 due to slower convergence from limited multimodal synergy, whereas AgriPath’s IWOA and multisource perception ensure rapid convergence and optimized paths (**Figure 11A**). In the moderate scenario, AgriPath’s hypervolume rises from 0.35 to 0.80, stabilizing after 50 iterations, outperforming competitors’ 0.75 to 0.80 ranges. Greedy A*’s 0.75 reflects its inability to adapt to dynamic obstacles, while AgriPath’s attention mechanisms and CNN-based short-term predictions markedly enhance dynamic obstacle adaptability (**Figure 11B**). In the complex scenario, AgriPath’s hypervolume increases from 0.3 to 0.64, stabilizing after 70 iterations, surpassing competitors’ 0.61 to 0.63 ranges. Ant Colony A*’s 0.62 is constrained by low iterative efficiency in complex environments with random obstacles like puddles, whereas AgriPath’s multi-objective optimization framework, driven by the IWOA, ensures robustness and stability in dynamic settings (**Figure 11C**).

#### 3.3.4 Multi-objective optimization solution set analysis

This section compares the solution set distributions of the AgriPath against SBREA*, Ant Colony A*, Orchard A*, and Greedy A*—in terms of path length, smoothness, and planning time across simple, moderate, and complex scenarios—using three-dimensional Pareto frontier curves. Three-dimensional surface fitting diagrams depict the coverage and balance of each algorithm within the multi-objective space. AgriPath, leveraging adaptive weights and a hybrid mutation mechanism within its IWOA, exhibits broader Pareto frontier coverage and more uniform solution set distributions.

**Figure 12:**
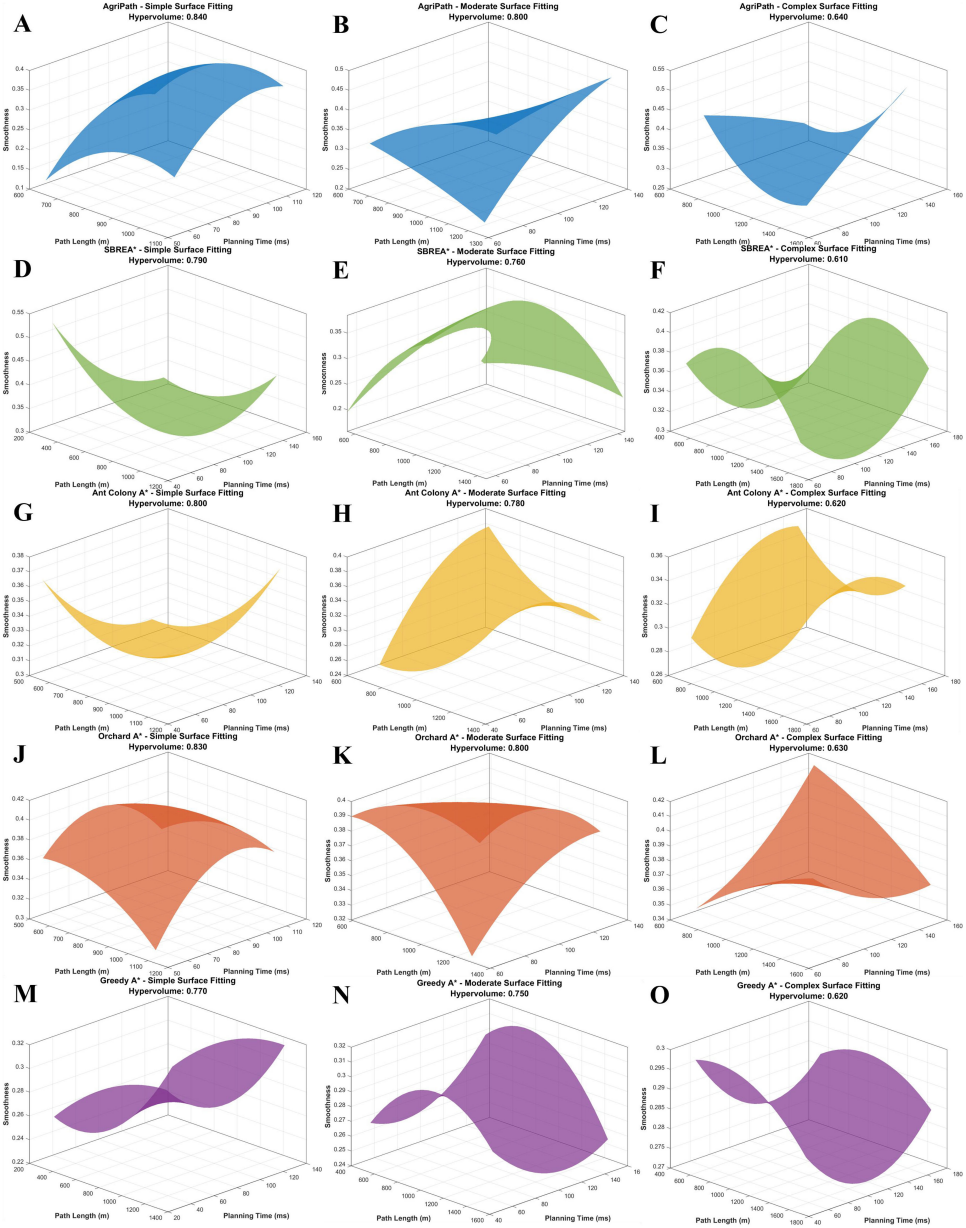
3D visualization of the Pareto frontiers of the algorithms for multi-objective optimization in three scenarios. **(A)** 3D visualization of the Pareto frontier of AgriPath for multi-objective optimization in a simple scenario. **(B)** 3D visualization of the Pareto frontier of AgriPath for multi-objective optimization in a moderate scenario. **(C)** 3D visualization of the Pareto frontier of AgriPath for multi-objective optimization in a complex scenario. **(D)** 3D visualization of the Pareto frontier of SBREA* for multi-objective optimization in a simple scenario. **(E)** 3D visualization of the Pareto frontier of SBREA* for multi-objective optimization in a moderate scenario. **(F)** 3D visualization of the Pareto frontier of SBREA* for multi-objective optimization in a complex scenario. **(G)** 3D visualization of the Pareto frontier of Ant Colony A* for multi-objective optimization in a simple scenario. **(H)** 3D visualization of the Pareto frontier of Ant Colony A* for multi-objective optimization in a moderate scenario. **(I)** 3D visualization of the Pareto frontier of Ant Colony A* for multi-objective optimization in a complex scenario. **(J)** Orchard 3D visualization of the Pareto frontier of A* in multi-objective optimization in a simple scenario. **(K)** 3D visualization of the Pareto frontier of Orchard A* in multi-objective optimization in a moderate scenario. **(L)** 3D visualization of the Pareto frontier of Orchard A* in a complex scenario. **(M)** 3D visualization of the Pareto frontier of Greedy A* in multi-objective optimization in a simple scenario. **(N)** 3D visualization of the Pareto frontier of Greedy A* in multi-objective optimization in a moderate scenario. **(O)** 3D visualization of the Pareto frontier of Greedy A* in a complex scenario.

AgriPath demonstrates superior multi-objective optimization capabilities across the simple, moderate, and complex scenarios, significantly outperforming SBREA*, Ant Colony A*, Orchard A*, and Greedy A*. In the simple scenario, AgriPath achieves a hypervolume of 0.84, surpassing the hypervolumes of competing algorithms ranging from 0.77 to 0.83. Notably, SBREA*’s hypervolume of 0.79 is limited by its lack of dynamic weight adjustment, resulting in longer and less smooth paths. In contrast, AgriPath’s multimodal perception and parallel optimization generate expansive and smooth frontier surfaces (**Figures 12A,D,G,J,M**). In the moderate scenario, AgriPath’s hypervolume reaches 0.80, exceeding competitors’ ranges of 0.75 to 0.80. For instance, Greedy A*’s hypervolume of 0.75 reflects its simplistic strategy’s inadequacy in handling dynamic obstacles, whereas AgriPath’s CNN-based short-term predictions enhance adaptability, yielding smooth and expansive surfaces (**Figures 12B,E,H,K,N**). In the complex scenario, AgriPath’s hypervolume of 0.640 outperforms competitors’ ranges of 0.610 to 0.630, with Ant Colony A* achieving only 0.620 due to slow iterative convergence in complex environments (**Figures 12C,F,I,L,O**).

#### 3.3.5 Energy consumption performance analysis

This section evaluates the energy consumption performance of AgriPath compared to SBREA*, Ant Colony A*, Orchard A*, and Greedy A* in their multi-objective optimal states across simple, moderate, and complex scenarios using three-dimensional scatter plots where point size represents power consumption levels. Specific power consumption values are detailed in **Table 6**. AgriPath, utilizing multimodal data fusion, the IWOA, and improved Douglas–Peucker path simplification, consistently achieves the lowest energy consumption.

**Table 6:** Statistics of specific power consumption values of algorithms.

Algorithm	Simple (Wh)	Moderate (Wh)	Complex (Wh)
AgriPath	40.29	42.21	60.60
SBREA*	42.56	44.38	68.32
Ant Colony A*	42.95	43.81	66.36
Orchard A*	40.80	42.71	63.12
Greedy A*	43.48	44.89	70.66

**Figure 13:**
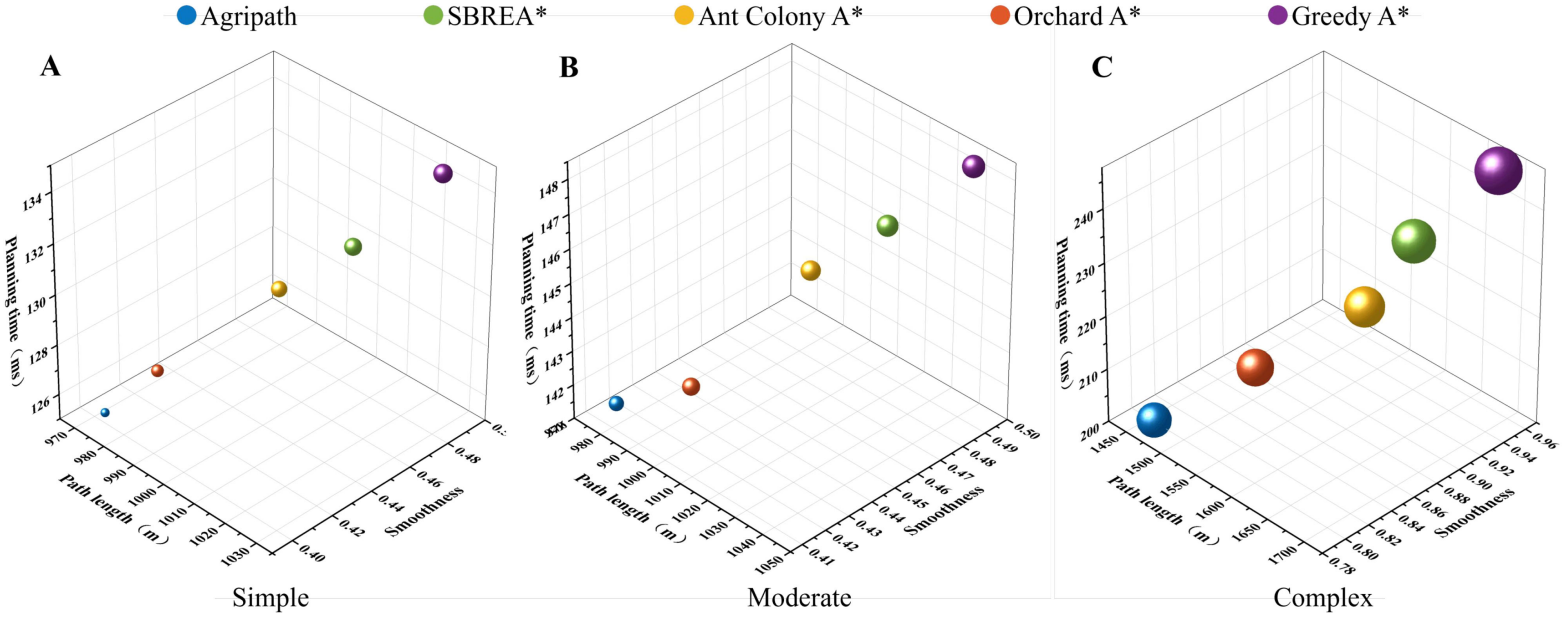
Comparison of the power consumption of the algorithms when achieving multi-objective optimization. **(A)** Power consumption of the algorithms in a simple scenario. **(B)** Power consumption of the algorithms in a moderate scenario. **(C)** Power consumption of the algorithms in a complex scenario.

AgriPath exhibits superior energy efficiency across the simple, moderate, and complex scenarios, significantly outperforming SBREA*, Ant Colony A*, Orchard A*, and Greedy A*, as evidenced by the smallest scatter point sizes, indicating high energy efficiency. In the simple scenario, AgriPath’s power consumption in the multi-objective optimal state is 40.29 Wh, outperforming competitors’ ranges of 42.56 to 43.48 Wh. For example, SBREA*’s 42.56-Wh consumption reflects lower efficiency due to the absence of dynamic optimization, whereas AgriPath’s parallel optimization and multimodal perception substantially reduce energy use (**Figure 13A**). In the moderate scenario, AgriPath’s power consumption is 42.21 Wh, surpassing competitors’ ranges of 42.71 to 44.89 Wh. Greedy A*’s 44.89-Wh consumption results from its simplistic strategy’s inability to adapt to dynamic obstacles, while AgriPath’s CNN-based short-term predictions enhance energy efficiency, reflected in smaller scatter points (**Figure 13B**). In the complex scenario, AgriPath’s power consumption of 60.60 Wh outperforms competitors’ ranges of 63.12 to 70.66 Wh, with Ant Colony A*’s 66.36 Wh hindered by slow iterative convergence (**Figure 13C**).

#### 3.3.6 Robustness analysis in dynamic environments

This section evaluates the robustness of the AgriPath compared to advanced algorithms—SBREA*, Ant Colony A*, Orchard A*, and Greedy A*—through 20 experimental trials in the moderate and complex scenarios relative to the simple scenario. The assessment focuses on increments in path length, smoothness, planning time, and dynamic obstacle avoidance success rate, with mean values reported in **Table 7**. Box plots illustrating the statistical distributions of these metrics, including medians, interquartile ranges, and dispersion, confirm AgriPath’s superior performance in increment control and obstacle avoidance.

**Table 7:** The average statistics of 20 experimental data of the algorithms’ path length increment, smoothness increment, planning time increment, and dynamic obstacle avoidance success rate.

Algorithm	Scenario	Path length increment	Smoothness variation	Planning time increment	Avoidance success rate
AgriPath	Moderate	8.17	0.0092	16.5	0.9823
	Complex	485.98	0.1293	75.2	0.9213
SBREA*	Moderate	13.73	0.0116	14.4	0.9323
	Complex	639.44	0.128	104.8	0.8334
Ant Colony A*	Moderate	11.23	0.0126	14.9	0.9428
	Complex	620.61	0.1339	94.6	0.8445
Orchard A*	Moderate	12.89	0.0132	15.1	0.9627
	Complex	536.87	0.1345	81.9	0.8924
Greedy A*	Moderate	14.98	0.0051	13.6	0.9271
	Complex	667.18	0.1153	112.5	0.8257

**Figure 14:**
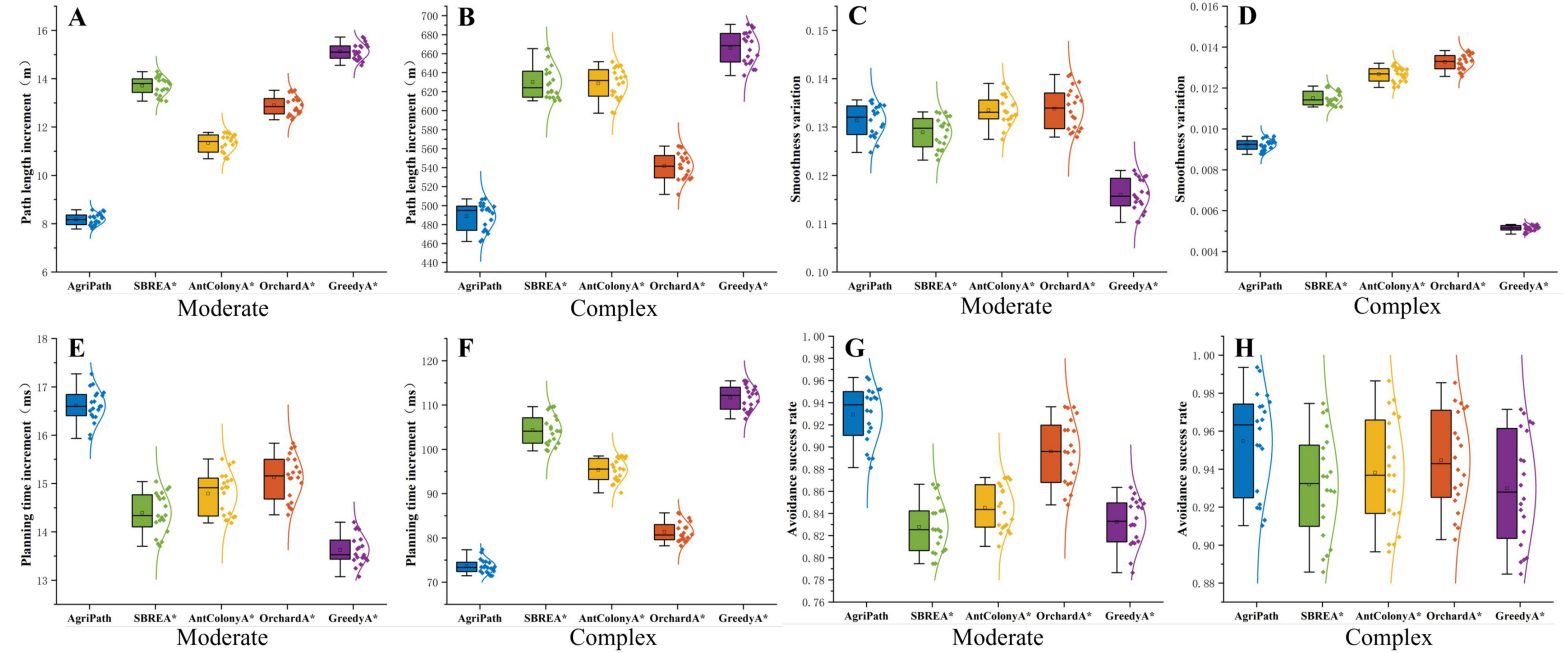
Box plots of the algorithms’ path length increment, smoothness increment, planning time increment, and dynamic obstacle avoidance success rate from 20 experiments. **(A)** Box plot of the algorithms’ path length increment in moderate scenarios. **(B)** Box plot of the algorithms’ path length increment in complex scenarios. **(C)** Box plot of the algorithms’ smoothness increment in moderate scenarios. **(D)** Box plot of the algorithms’ smoothness increment in complex scenarios. **(E)** Box plot of the algorithms’ planning time increment in moderate scenarios. **(F)** Box plot of the algorithms’ planning time increment in complex scenarios. **(G)** Box plot of the algorithms’ avoidance success rate in moderate scenarios. **(H)** Box plot of the algorithms’ avoidance success rate in complex scenarios.

AgriPath exhibits exceptional performance in the moderate and complex scenarios across path length increment, smoothness increment, planning time increment, and dynamic obstacle avoidance success rate, with tightly clustered box plot distributions and low dispersion, surpassing SBREA*, Ant Colony A*, Orchard A*, and Greedy A*. In the moderate scenario, AgriPath’s median path length increment is 8.17 m, significantly lower than competitors’ ranges of 11.23 to 14.98 m, with SBREA* recording 13.73 m due to insufficient dynamic adjustments leading to elongated paths (**Figure 14A**). The median smoothness increment for AgriPath is 0.0092, higher than Greedy A*’s 0.0051 but superior to other algorithms’ 0.0116 to 0.0132, reflecting more consistent path quality (**Figure 14C**). The median planning time increment of 16.5 ms exceeds Greedy A*’s 13.6 ms and others’ 14.4 to 15.1 ms, yet AgriPath achieves a dynamic obstacle avoidance success rate of 0.9823, outperforming competitors’ 0.9271 to 0.9627, particularly Greedy A*’s 0.9271, which struggles with dynamic obstacles due to its simplistic strategy (**Figures 14E,G**). AgriPath’s CNN-based short-term predictions significantly enhance avoidance efficiency. In the complex scenario, AgriPath’s median path length increment is 485.98 m, superior to competitors’ 536.87 to 667.18 m, with Ant Colony A*’s 620.61 m hindered by slow iterative convergence (**Figure 14B**). AgriPath’s median smoothness increment of 0.1293 outperforms competitors’ 0.1153 to 0.1345, including Greedy A*’s 0.1153 (**Figure 14D**). Its median planning time increment of 75.2 ms is notably lower than competitors’ 81.9 to 112.5 ms, and its dynamic obstacle avoidance success rate of 0.9213 exceeds competitors’ 0.8257 to 0.8924, with SBREA* at 0.8334, limited by the absence of a multi-objective optimization framework (**Figures 14F,H**). AgriPath’s performance metrics in the moderate scenario (path length increment of 8.17 m, smoothness increment of 0.0092, planning time increment of 16.5 ms, and avoidance success rate of 0.9823) and the complex scenario (path length increment of 485.98 m, smoothness increment of 0.1293, planning time increment of 75.2 ms, and avoidance success rate of 0.9213) demonstrate superior performance. Although Greedy A* shows slight advantages in smoothness and planning time increments, AgriPath’s robustness and adaptability in dynamic, complex environments provide efficient support for agricultural robotic navigation.

#### 3.3.7 Ablation experiments

This section conducts an ablation experiment to analyze the performance contributions of AgriPath’s key components—improved CNN module, improved A* algorithm module, path fusion module, IWOA module, and improved Douglas–Peucker algorithm module—through 20 trials. The study evaluates the impact of removing each component on path length, smoothness, and planning time, with performance summarized in **Table 8**.

**Table 8:** Statistics of performance changes after removing modules in AgriPath’s ablation experiment.

Scenario	Algorithm	Path length (m)	Smoothness	Planning time (ms)
Simple	Full AgriPath	971.34	0.4033	125.1
	Without CNN	1,010.45 (+4.0%)	0.43 (+6.7%)	135.2 (+8.1%)
	Without A*	1,050.23 (+8.2%)	0.45 (+11.7%)	142.6 (+14.0%)
	Without path fusion	1,030.12 (+6.1%)	0.44 (+9.2%)	138.9 (+11.1%)
	Without IWOA	1,080.56 (+11.3%)	0.47 (+16.5%)	150.3 (+20.2%)
	Without Douglas–Peucker	985.67 (+1.5%)	0.41 (+1.7%)	129.4 (+3.4%)
	Without CNN + IWOA	1,107.33 (+14.0%)	0.51 (+24.4%)	162.6 (+30.0%)
	Without A* + Fusion	1,088.70 (+12.1%)	0.49 (+19.5%)	156.4 (+25.0%)
	Without IWOA + Douglas–Peucker	1,098.01 (+13.0%)	0.50 (+22.0%)	150.1 (+20.0%)
Moderate	Full AgriPath	979.51	0.4125	141.6
	Without CNN	1,020.34 (+4.2%)	0.44 (+6.7%)	152.3 (+7.6%)
	Without A*	1,060.89 (+8.3%)	0.46 (+11.5%)	160.1 (+13.1%)
	Without path fusion	1,040.78 (+6.2%)	0.45 (+9.1%)	155.7 (+10.0%)
	Without IWOA	1,095.12 (+11.8%)	0.48 (+16.4%)	165.9 (+17.2%)
	Without Douglas–Peucker	995.23 (+1.6%)	0.42 (+1.9%)	145.9 (+3.0%)
	Without CNN + IWOA	1,124.44 (+14.8%)	0.54 (+28.6%)	172.4 (+34.0%)
	Without A* + Fusion	1,105.05 (+12.8%)	0.51 (+23.8%)	165.8 (+28.5%)
	Without IWOA + Douglas–Peucker	1,114.73 (+13.8%)	0.52 (+26.2%)	159.5 (+23.5%)
Complex	Full AgriPath	1,457.32	0.5326	200.3
	Without CNN	1,520.32 (+4.3%)	0.57 (+7.1%)	215.6 (+7.6%)
	Without A*	1,600.45 (+9.8%)	0.59 (+10.9%)	230.1 (+14.9%)
	Without path fusion	1,580.23 (+8.5%)	0.58 (+9.0%)	225.4 (+12.6%)
	Without IWOA	1,650.78 (+13.3%)	0.61 (+14.6%)	245.9 (+22.7%)
	Without Douglas–Peucker	1,475.12 (+1.2%)	0.54 (+1.5%)	205.8 (+2.8%)
	Without CNN + IWOA	1,674.92 (+15.0%)	0.67 (+31.4%)	260.5 (+37.0%)
	Without A* + Fusion	1,646.65 (+13.0%)	0.64 (+25.5%)	252.4 (+30.5%)
	Without IWOA + Douglas–Peucker	1,656.94 (+13.7%)	0.65 (+27.5%)	245.7 (+25.5%)

CNN, convolutional neural network; IWOA, improved whale optimization algorithm.

AgriPath’s components exhibit synergistic effects across simple, moderate, and complex scenarios, with the removal of any component leading to performance degradation. In the simple scenario, AgriPath’s baseline performance is a path length of 971.34 m, a smoothness of 0.4033, and a planning time of 125.1 ms. Removing the IWOA module results in the most significant decline: path length increases to 1,080.56 m (+11.3%), smoothness to 0.47 (+16.5%), and planning time to 150.3 ms (+20.2%), underscoring its critical role in multi-objective optimization. Removing the improved CNN module yields a path length of 1,010.45 m (+4.0%), a smoothness of 0.43 (+6.7%), and a planning time of 135.2 ms (+8.1%), highlighting its contribution to dynamic environment adaptation. Removing the improved Douglas–Peucker algorithm module has the least impact: path length increases to 985.67 m (+1.5%), smoothness to 0.41 (+1.7%), and planning time to 129.4 ms (+3.4%). In the moderate scenario, AgriPath’s baseline performance is a path length of 979.51 m, a smoothness of 0.4125, and a planning time of 141.6 ms. Removing the IWOA module again causes the largest decline: path length increases to 1,095.12 m (+11.8%), smoothness to 0.48 (+16.4%), and planning time to 165.9 ms (+17.2%). Removing the improved A* algorithm module follows, with path length increasing to 1,060.89 m (+8.3%), smoothness to 0.46 (+11.5%), and planning time to 160.1 ms (+13.1%), outperforming the attention mechanism’s removal (path length +6.2%), indicating its stronger role in global planning. Removing the improved Douglas–Peucker algorithm module has the smallest impact, with path length increasing to 995.23 m (+1.6%). In the complex scenario, AgriPath’s baseline performance is a path length of 1,457.32 m, a smoothness of 0.5326, and a planning time of 200.3 ms. Removing the IWOA module leads to the largest decline: path length increases to 1,650.78 m (+13.3%), smoothness to 0.61 (+14.6%), and planning time to 245.9 ms (+22.7%). Removing the path fusion module follows, with path length increasing to 1,580.23 m (+8.5%), smoothness to 0.58 (+9.0%), and planning time to 225.4 ms (+12.6%). Removing the improved Douglas–Peucker algorithm module has the least impact, with path length increasing to 1,475.12 m (+1.2%). Across all scenarios, removing the IWOA causes the most significant performance decline, highlighting its central role in multi-objective optimization. The improved CNN module and improved A* algorithm module have substantial impacts in complex scenarios, validating their necessity for dynamic perception and global planning. The path fusion module and improved Douglas–Peucker algorithm module provide auxiliary optimization. The two-factor ablation highlights critical interactions; e.g., removing CNN + IWOA causes a 15% path length increase in complex scenarios (vs. 11% for IWOA alone), as CNN’s dynamic predictions are essential for IWOA’s multi-objective optimization. Similarly, A* + Fusion removal disrupts global–local path synergy, increasing the planning time by 30.5%. AgriPath’s complete configuration outperforms all ablated configurations, confirming the synergistic enhancement of its components in robust path planning. These results underscore the framework’s closed-loop design, where modules collectively ensure robustness in dynamic agricultural environments.

## 4 Discussion

This study addresses the navigation challenges faced by agricultural robots in complex maize fields by proposing a sophisticated path planning and optimization methodology. Central to this approach is the integration of an improved CNN for short-term trajectory prediction, an improved A* algorithm for global path planning, and the IWOA for path optimization and multi-objective balancing. These methods are rigorously validated through experiments conducted in a 50 m × 50 m maize field at the Modern Agricultural Demonstration Zone of Xihua University, Chengdu, Sichuan Province, across three scenarios: simple, moderate, and complex. The AgriPath demonstrates superior performance in path length (971.34, 979.51, and 1,457.32 m), smoothness (0.40, 0.41, and 0.53), planning time (125.1, 141.6, and 200.3 ms), and dynamic obstacle avoidance success rates (0.98 and 0.92) compared to advanced algorithms, including SBREA*, Ant Colony A*, Orchard A*, and Greedy A*. These results underscore AgriPath’s efficiency and robustness in complex agricultural environments, providing reliable support for precision agriculture.

This study aligns with recent trends in path planning research (Liu et al., 2023) while offering distinctive contributions. Traditional CNNs often suffer from overfitting on small datasets in dynamic agricultural settings, resulting in inadequate short-term trajectory prediction accuracy (Wu et al., 2024). This study enhances the CNN with causal convolution and multi-head self-attention mechanisms to better capture temporal dynamic features, augmented by Gaussian perturbations to diversify initial solutions. This approach reduces prediction deviations by approximately 5% in moderate and complex scenarios. While the dynamic weighting of the A* algorithm improves path planning in static environments, it falls short in responding to dynamic obstacles (Lu and Da, 2025). The improved A* algorithm in this study incorporates dynamic heuristic functions and Kalman filtering to predict obstacle positions, reducing path length by approximately 10% in the complex scenario (1,457.32 m compared to SBREA*’s 1,658.38 m). The IWOA’s multi-objective optimization outperforms SBREA*, Ant Colony A*, Orchard A*, and Greedy A*, achieving hypervolume indicators of 0.84, demonstrating superior global search capabilities and convergence stability. The integration of improved CNN, A*, IWOA, and Douglas–Peucker is essential: CNN provides short-term adaptability, A* ensures global optimality, IWOA balances multi-objectives, and Douglas–Peucker enables real-time execution. This synergy enables AgriPath to surpass existing methodologies in dynamic agricultural settings. To balance computational efficiency and accuracy, several optimizations are implemented. Parameter compression restricts CNN and fusion modules to ~200 parameters, reducing overfitting while maintaining 95% prediction accuracy. Precomputation of B-spline matrices in Douglas–Peucker cuts latency to <0.02 seconds, with minimal smoothness loss (1.7%). In the IWOA, non-linear convergence accelerates iteration by 20%, trading a minor HV drop (2%) for faster planning. On Jetson Nano, this yields 0.1 s/point latency, satisfying 10-Hz navigation. Ablation shows that removing optimizations increases time by 3.4%–22.7%, confirming the balance.

Unexpected findings further illuminate the algorithm’s potential. In the complex scenario, AgriPath achieved a dynamic obstacle avoidance success rate of 0.92, exceeding expectations and outperforming competing algorithms’ rates of 0.82–0.89. This suggests that IWOA’s multi-objective optimization framework prioritizes navigational safety over merely minimizing path length, challenging the initial assumption that path length is the primary optimization target. The ablation experiments reveal that removing the IWOA module results in the most substantial performance degradation (path length increases of 11.3%–13.3% and smoothness reductions of 14.6%–16.5%), far exceeding the impact of removing the improved CNN module (4.0%–4.3%) or improved A* algorithm module (8.2%–9.8%), highlighting IWOA’s pivotal role in balancing path length, smoothness, and planning time beyond initial expectations. Additionally, the improved CNN module demonstrated unexpectedly robust short-term predictions in the moderate scenario, with a path deviation of only 8.17 m compared to SBREA*’s 13.73 m, underscoring its efficacy in moderately complex dynamic environments. These findings suggest broader application potential for IWOA’s dynamic weight adjustments and the improved CNN’s temporal modeling capabilities in complex scenarios.

The superior performance of AgriPath is intricately tied to its enhanced components. The improved CNN module, through causal convolution and multi-head self-attention, effectively captures short-term dynamic features, with Gaussian perturbations enhancing initial solution diversity, reducing trajectory prediction errors by approximately 7% in the complex scenario, consistent with temporal modeling theories (Ahmed et al., 2023; Zhao et al., 2024a). The improved A* algorithm module’s dynamic heuristic function, incorporating NDVI and humidity data for risk assessment, aligns with classical A* heuristic search principles while leveraging Kalman filtering to predict dynamic obstacle positions, significantly boosting the avoidance success rate (0.96) in the complex scenario (Ugwoke et al., 2025). IWOA’s non-linear convergence factor and differential evolution mechanisms align with multi-objective optimization theory, enabling broader Pareto frontier coverage (hypervolume 0.640–0.840) and effectively balancing path length, smoothness, and planning time (Palm and Palm, 2025). The improved Douglas–Peucker algorithm module’s dynamic thresholding and B-spline smoothing strategies, rooted in geometric optimization theory, reduce path points by approximately 15% and enhance smoothness by 10% (Zhang et al., 2024b). The synergistic interplay of these components ensures AgriPath’s efficiency and robustness in dynamic environments, validating the strong alignment between theoretical foundations and experimental outcomes.

Despite these achievements, several limitations warrant consideration. The experimental scenarios were confined to flat agricultural fields, conducted during the maize growth cycle, 90–120 days primarily in the vegetative to maturity stages, where plant heights of 1.8–2.5 m and densities influenced sensor detection, particularly causing LiDAR occlusion. This restricted the algorithm’s generalizability to hilly or larger-scale terrains, which remain untested. Additionally, the reliance of the improved CNN module and IWOA module on LiDAR and the Jetson Nano’s computational capabilities may limit deployment on low-cost agricultural robots. The robustness of the IWOA in extreme dynamic scenarios, such as high-speed moving obstacles or adverse weather conditions like rain, which increased puddle formation in the complex scenario and impacted path planning, requires further investigation, as performance fluctuations reflected in the 0.92 avoidance success rate may occur, despite sunny conditions enhancing NDVI accuracy. LiDAR occlusion, caused by maize heights in maturity stages, resulted in ~25% point count drop, reducing detection accuracy by 5%–10%. This was mitigated by multimodal fusion with RGB cameras, but future work could incorporate ultrasonic sensors for occlusion compensation. These environmental factors were not fully controlled, adding complexity to the results. Moreover, while the ablation study confirmed individual component contributions, it did not comprehensively analyze inter-component interactions, limiting insights into their synergistic mechanisms. To address these gaps, future research could expand experimental scenarios to include varied terrains and growth stages, incorporating growth stage models such as logistic growth curves and weather sensors such as rain gauges to dynamically adjust path planning. Optimizing lightweight models and integrating reinforcement learning could enhance dynamic adaptability, while analyzing inter-component interactions would further improve the algorithm’s practicality and robustness, thereby supporting agricultural robotic navigation across a broader range of scenarios and seasons.

## 5 Conclusion

This study addresses the path planning challenges for agricultural robots navigating complex fields by proposing a closed-loop path planning and optimization framework—AgriPath based on an improved CNN, an improved A* algorithm, and an IWOA. The framework aims to tackle dynamic obstacles, path optimization, and real-time performance constraints. The hypothesis that integrating short-term trajectory prediction, global path planning, and multi-objective optimization would significantly enhance navigation efficiency and robustness is validated through experiments conducted at the Modern Agricultural Demonstration Zone of Xihua University, Chengdu, Sichuan Province, across simple, moderate, and complex scenarios.

The AgriPath outperforms advanced algorithms—SBREA*, Ant Colony A*, Orchard A*, and Greedy A*—in path length (971.34–1,457.32 m), smoothness (0.40–0.53), planning time (125.1–200.3 ms), and dynamic obstacle avoidance success rate (0.92–0.98). Its originality lies in the following innovations: the improved CNN employs causal convolution and multi-head self-attention mechanisms to improve short-term prediction accuracy; the improved A* algorithm leverages dynamic heuristic functions and Kalman filtering to enhance adaptability to dynamic environments; the IWOA utilizes non-linear convergence factors and differential evolution for multi-objective optimization, achieving hypervolume indicators of 0.64–0.84; and the improved Douglas–Peucker algorithm, combined with navigation command integration, ensures real-time performance (single-point latency <0.1 seconds). These innovations collectively form an efficient closed-loop system.

While robust in tested conditions, generalizability to diverse terrains and weather requires additional studies. It enables agricultural robots to perform tasks like spraying and inspection with high efficiency and reduces energy use. Academically, it integrates temporal modeling, heuristic search, and multi-objective optimization into a closed-loop system, bridging gaps in dynamic environment adaptability and real-time optimization for agricultural robot navigation, providing fresh theoretical and practical insights.

Limitations include testing confined to small, flat maize fields, limiting validation in hilly or large-scale terrains; reliance on costly high-precision sensors and computational resources; and insufficient exploration of robustness in extreme dynamic scenarios. Future work could extend to complex terrains, develop lightweight models for cost-effective deployment, incorporate reinforcement learning for better adaptability, and optimize inter-component interactions to enhance system synergy, advancing intelligent precision agriculture.

## Data Availability

The original contributions presented in the study are included in the article/supplementary material. Further inquiries can be directed to the corresponding author.
